# Molecular, Pathological, Clinical, and Therapeutic Aspects of Perihematomal Edema in Different Stages of Intracerebral Hemorrhage

**DOI:** 10.1155/2022/3948921

**Published:** 2022-09-17

**Authors:** Chao Jiang, Hengtao Guo, Zhiying Zhang, Yali Wang, Simon Liu, Jonathan Lai, Tom J. Wang, Shize Li, Jing Zhang, Li Zhu, Peiji Fu, Jiewen Zhang, Jian Wang

**Affiliations:** ^1^Department of Neurology, The Fifth Affiliated Hospital of Zhengzhou University, 450052 Zhengzhou, Henan, China; ^2^Medical Genomics Unit, National Human Genome Research Institute, Bethesda, MD 20814, USA; ^3^Baylor University, Waco, Texas 76706, USA; ^4^The Johns Hopkins University, Baltimore, MD 21218, USA; ^5^Department of Neurology, Zhengzhou Central Hospital Affiliated to Zhengzhou University, 450001 Zhengzhou, Henan, China; ^6^Department of Neurology, People's Hospital of Zhengzhou University, 450003 Zhengzhou, Henan, China; ^7^Department of Anatomy, School of Basic Medical Sciences, Zhengzhou University, 450001 Zhengzhou, Henan, China

## Abstract

Acute intracerebral hemorrhage (ICH) is a devastating type of stroke worldwide. Neuronal destruction involved in the brain damage process caused by ICH includes a primary injury formed by the mass effect of the hematoma and a secondary injury induced by the degradation products of a blood clot. Additionally, factors in the coagulation cascade and complement activation process also contribute to secondary brain injury by promoting the disruption of the blood-brain barrier and neuronal cell degeneration by enhancing the inflammatory response, oxidative stress, etc. Although treatment options for direct damage are limited, various strategies have been proposed to treat secondary injury post-ICH. Perihematomal edema (PHE) is a potential surrogate marker for secondary injury and may contribute to poor outcomes after ICH. Therefore, it is essential to investigate the underlying pathological mechanism, evolution, and potential therapeutic strategies to treat PHE. Here, we review the pathophysiology and imaging characteristics of PHE at different stages after acute ICH. As illustrated in preclinical and clinical studies, we discussed the merits and limitations of varying PHE quantification protocols, including absolute PHE volume, relative PHE volume, and extension distance calculated with images and other techniques. Importantly, this review summarizes the factors that affect PHE by focusing on traditional variables, the cerebral venous drainage system, and the brain lymphatic drainage system. Finally, to facilitate translational research, we analyze why the relationship between PHE and the functional outcome of ICH is currently controversial. We also emphasize promising therapeutic approaches that modulate multiple targets to alleviate PHE and promote neurologic recovery after acute ICH.

## 1. Introduction

Intracerebral hemorrhage (ICH) is caused by the spontaneous rupture of small penetrating arteries or arterioles in the brain parenchyma [1, 2]. Although it only accounts for 15-20% of total stroke incidence, ICH has a high rate of mortality and disability [1, 3]. More than 3 million patients worldwide develop ICH annually, with an incidence of approximately 24.68 per 100,000 [3–5]. The global burden induced by ICH is heavy now [6]. Once ICH occurs, blood will leak into the brain parenchyma, forming a hematoma that compresses the brain and leads to neurologic deficits [3, 4, 7]. In addition to the mass effect of the hematoma associated with the primary injury, the subsequent coagulation cascade and the degradation of the hematoma lead to secondary damage to the surrounding brain parenchyma in the following days or weeks [8–10]. These extra injuries can further aggravate neurologic deficits after ICH. Furthermore, the mass effect of the hematoma combined with the crushing impact of neurotoxicity-induced brain swelling in the perihematomal areas can also increase intracranial pressure (ICP), even leading to cerebral herniation or death [3, 4]. Therefore, there is a pressing need to explore new approaches to mitigate secondary brain damage after acute ICH due to the limited benefits of surgical removal of hematomas [3, 11].

Perihematomal edema (PHE), which is referred to as increases in water content in brain tissue adjacent to intraparenchymal hematoma, may represent a potential surrogate marker or a simple imaging indicator to assess secondary brain damage after ICH [12]. It has been used to assess the efficacy of the intervention as an early endpoint indicator, as shown in phase 1 and phase 2 clinical trials. However, clinicians should carefully evaluate its utility, and limitations for its clinical importance warrant further verification [13, 14]. Although there are multiple methods to quantify PHE, their reliability has not been thoroughly evaluated. In addition, the predictive value of PHE on the prognosis of ICH seems also to be inconsistent and controversial. Therefore, it is critical to better understand the reliability of PHE quantification methods and the clinical significance of PHE after ICH. So far, we know that PHE develops and progresses in several stages, each of which has significant morphological differences along with the corresponding molecular changes [13–17]. Evidence indicates that cerebral edema, predominantly neuronal cytotoxic edema, is not irreversible in its early phase after stroke [18, 19]. Before we can establish the causal relationship between the severity of PHE and the functional outcomes of ICH, more studies are needed to investigate the mechanism of PHE to identify therapeutic targets. A deep understanding of the etiological factors that influence the formation and progression in different stages of PHE can help identify potential therapeutic targets.

In this review, we will summarize recent advances in the staging, classification, mechanism, imaging characteristics, and quantification of PHE. Furthermore, we will discuss the variables that may affect the severity of PHE and the relationship between PHE and the functional results of ICH. Finally, we will also summarize the potential therapeutic targets for PHE. Previously, the underlying mechanisms of PHE in different stages after ICH have been documented. To increase the clinical significance of PHE, in this article, we include the imaging characteristics and evolution of ionic edema and vasogenic edema in the perihematomal areas. We emphasized the usages and limitations of different quantitative methods of PHE in preclinical and clinical studies. We also reviewed the influence of baseline variables and laboratory and clinical variables on PHE in animals and humans, including imaging characteristics, cerebral venous drainage system, and brain lymphatic drainage system. To promote clinical transformation, we explain the reasons for the current conflict results in the relationship between PHE and ICH functional outcomes of ICH in this article. We analyze which PHE quantitation method may be more promising or reliable for ICH prediction. In preclinical and clinical studies, we also reviewed the therapeutic advances for PHE by emphasizing dehydration therapy, clot or RBC clearance, hemostasis and iron chelation, blood pressure control, immunomodulatory therapy, etc. This review will help highlight the clinical importance of PHE after acute ICH and identify potential therapeutic targets to facilitate translational research.

## 2. The Time Phase and Pathophysiology of PHE

The natural evolution of PHE involves increases in the volume of edema up to 14 days or even longer in patients with ICH [20, 21]. Furthermore, the formation and evolution of PHE are complex and involve multiple ICH-induced pathophysiological pathways [12]. The coagulation cascade and hematolysis contribute to the formation of PHE [14, 22]. We will discuss the factors involved in the coagulation cascade and the hematolysis response in the pathophysiology of PHE. The evolution of PHE occurs in three distinct phases, the pathophysiology of which is summarized in Table 1.

### 2.1. Hyperacute Phase

Brain edema occurs around the hematoma a few hours after ICH [14, 15]. The pathophysiology of PHE in the hyperacute phase of ICH has not been fully elucidated. Animal research in pigs has revealed that the white matter adjacent to the hematoma had a greater than 10% increase in water content (>85%) when compared to the contralateral white matter (73%) as early as one hour after ICH [23]. The increase in water content persisted throughout the observed 8 hours after ICH [23]. Furthermore, fibrinogen extrusion increased significantly in one hour in these areas, while damage to the blood-brain barrier (BBB) was not critical during that time frame [23]. Interestingly, another study in rats also revealed a quantitative association between protein content in edema fluid and the rate of fluid clearance in the brain [24]. Therefore, rapid PHE formation can be induced primarily by an increase in interstitial osmotic pressure established by clot retraction and extrusion of serum protein from the hematoma in the hyperacute phase of ICH [14]. Although serum albumin extrusion can also promote cellular apoptosis [25], it is currently unknown whether it can cause additional brain damage in the hyperacute phase of ICH.

Regarding other possible explanations, evidence implies that the appearance of cytotoxic or ionic edema may be associated with extracellular accumulation of neurotoxins such as glutamate in the hyperacute phase of ICH, as these neurotoxins can induce severe and permanent neurotoxic damage [22, 25, 26]. Furthermore, a clinical study observed PHE with magnetic resonance imaging (MRI) in 56 of 83 patients with ICH within 6 hours after the onset of the disease. Subsequently, it attributed the restricted diffusion perihematomal area to transient oligemic and metabolic changes [27]. Using multimodal images including diffusion-weighted magnetic resonance imaging (DWI), apparent diffusion coefficient (ADC) maps, perfusion-weighted magnetic resonance imaging (PWI), and proton magnetic resonance spectroscopic (MRS) images, researchers also revealed that hypoperfusion in the perihematomal tissues might be caused by reduced metabolic demand (diaschisis) rather than ischemia [28–31]. Therefore, it is essential to explore whether a reduction in blood flow and energy metabolism (including mitochondrial dysfunction and Na-ATP pump failure) induced by the mass effect of the hematoma promotes the formation of PHE in the hyperacute phase.

### 2.2. Acute Phase

The activation of the coagulation cascade leads to clot formation by activating thrombin and fibrinogen, which play a role in the formation of PHE [14, 32]. Thrombin-induced PHE formation peaks at 1–2 days in the mouse brain [22, 33]. In this phase, cellular apoptosis or neuronal degeneration becomes increasingly evident in the perihematomal edema zone of ICH brain [34]. Studies have revealed that thrombin concentration was positively correlated with the severity of edema and neurologic impairment after acute ICH [14, 16]. Furthermore, thrombin inhibition significantly mitigated PHE evolution, suggesting that thrombin is the primary cause of early PHE [35]. The mechanism of thrombin-mediated PHE formation may be associated with an increase in BBB permeability after acute ICH [14]. The changes in the structure of the BBB are closely related to neuroinflammation after ICH [36]. There has been evidence that indicated that the combination of thrombin and protease-activated receptors (PAR) can upregulate the expression of inflammatory mediators (e.g., TNF-*α*, IL-1*β*, IL-6, IL-12, and ICAM), thus causing BBB disruption in the acute phase of ICH [37–40]. Other important factors mentioned as causes of vasogenic edema after ICH also include matrix metalloproteins (MMP) and aquaporin 4 (AQP4) [41, 42]. Unlike the effects induced by the change in water from the AQP4 channel, MMPs, including MMP-2, MMP-3, and MMP-9, mainly promote the formation of brain edema by degrading tight junctions and the basal lamina proteins of the BBB after ICH [41, 42]. There is also evidence that thrombin can directly disrupt the BBB by increasing the expression of MMPs and AQP4 *in vitro* and *in vivo* [43, 44]. Although it is a double-edged sword, VEGF may also tend to aggravate the vasogenic edema in the early stage of ICH [45, 46]. Additional evidence has indicated that thrombin can promote the release of vascular endothelial growth factor (VEGF) from astrocytes through the PAR-1/p44/42 mitogen-activated protein kinase (MAPK) pathway [47], suggesting that thrombin can also increase vascular permeability and subsequently promote the evolution of PHE by inducing VEGF overexpression.

Furthermore, thrombin can aggravate brain injury by activating the complement system after acute ICH [48, 49]. Evidence has implied that thrombin-cleaved C3a and C5a fragments increase inflammatory cell infiltration and aggravate PHE [50]. Moreover, complement-mediated ICH injury is associated with the membrane attack complex (MAC) [22]. MAC-mediated cell lysis and the inflammatory response further aggravate BBB leakage and promote PHE evolution [22]. The appearance of a stable clot depends on the interaction of thrombin, fibrinolysis factors, and platelets [51]. Research has revealed that FXa, a fibrinolysis factor, can also induce C3 cleavage, but no studies have investigated the influence of FXa on PHE in the acute phase of ICH [51]. Regarding platelets, there is evidence that platelet-derived growth factor receptor-*β* (TGF-*β*) can promote PHE by amplifying the inflammatory response in the perihematoma area [52]. In conjunction with the inflammatory response induced by the coagulation cascade, increased free radical generation promotes PHE formation [22]. Furthermore, the coagulation cascade associated with BBB breakdown can exacerbate the penetration of thrombin, fibrinolysis factors, platelets, and leukocyte recruitment from the blood circulation into brain tissue, further aggravating PHE [39, 40]. Therefore, it is essential to further explore the effects of the detailed mechanism of the coagulation cascade on PHE.

### 2.3. The Delayed Phase

Injection of red blood cells (RBC) into the brain has been shown to fail to induce brain edema in 24 hours, but an increase in brain edema appeared on day 3 and peaked on day 7 after injection [22]. Furthermore, neuronal degeneration and white matter injury in perihematomal tissues were prominent on day 3 or later post-ICH [53–55]. The above changes suggest that the toxicity of the RBC degradation products may aggravate brain injury by promoting the formation of acute edema on days 3-5 after ICH [22].

Studies have suggested that hemoglobin and its degradation products may play an essential role in delayed edema formation [14, 22, 56]. Hemoglobin, an erythrocyte lysis product, increased significantly during the first days after ICH [57–59]. Heme from hemoglobin is metabolized to iron, carbon monoxide, and biliverdin by heme oxygenase (HO) [60–63]. Evidence has indicated that hemoglobin, heme, ferrous iron, and carbon monoxide contribute to PHE formation and early brain injury [2, 22, 64, 65]. Although studies indicated that HO-2 exerted neuroprotective effects after ICH, HO-1 was identified with neurotoxic or angiogenic properties in acute or chronic phases [60, 61, 66–68]. Regarding the mechanisms related to delayed PHE formation, studies have revealed that hemoglobin and its degradation products promote cerebral edema by activating, among others, the oxidative stress response and the inflammatory response after acute ICH [22, 62].

Exceptional for the attraction and activation of leukocytes in the blood circulation, lysis products of RBC aggravate oxidative stress and inflammatory responses and promote delayed PHE formation by activating astrocytes and microglia [22, 63, 66, 69]. Although preclinical and clinical studies have explored the clearance of RBC lysis products on the evolution of PHE after ICH [57, 70–72], more studies on mechanisms related to the formation of delayed PHE will help identify potential therapeutic targets. Furthermore, additional studies on hemolysis imaging characteristics may benefit the prediction of the severity of PHE after ICH in different phases. Animal and clinical studies have revealed that hemolysis can present as a non-hypointense core in the heterogeneous background of hematomas on T2∗-weighted imaging [58, 59]. Furthermore, ICH-induced brain iron overload in the perihematomal area can also be quantified by R2∗ magnetic resonance mapping [73–75]. However, the above findings still need to be verified with histological evidence.

## 3. The Classification and Imaging Characteristics of PHE

**Figure 1:**
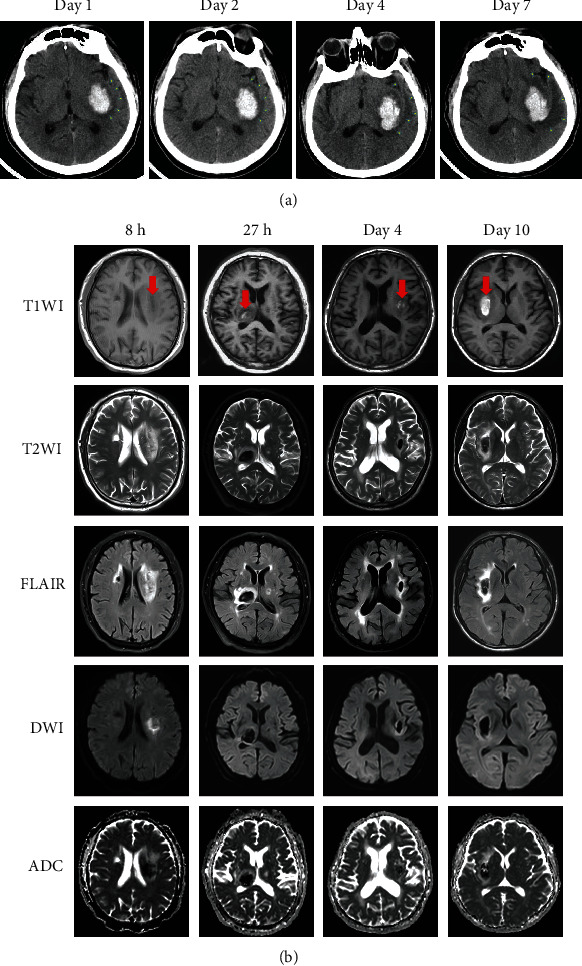
The imaging characteristics of PHE at different stages after ICH. (a) Cranial CT images on days 1, 2, 4, and 7 after acute ICH in a patient. PHE (a rim around the hematoma indicated by yellow arrows) was not prominent on day 1 after ICH and gradually increased from days 1 to 7 after ICH. (b) MRI characteristics of PHE at 8 hours, 27 hours, day 4, and day 10, respectively, after the onset of symptoms in 4 patients with ICH. The red arrows in the T1WI images indicate the location of the hematoma. The signal characteristics of the hematomas changed over time in the T1WI and T2WI images after ICH, with PHE presented as a thin or wide rim with a strong signal in the T2WI and FLAIR images in the areas surrounding the hematoma. A strong signal on the DWI image but a weak signal on the ADC map appeared in the perihematomal area 8 hours after ICH. It may represent the appearance of ionic edema or cytotoxic edema in the perihematomal area. However, typical imaging characteristics of vasogenic edema in the perihematomal region were observed at 27 hours, day 4, and day 10 after the onset of the symptom. Vasogenic edema presented as a normal signal on the DWI images, and a strong signal surrounds the hematoma on the ADC map at 27 hours, day 4, and day 10 after ICH in 3 patients. Abbreviations: CT: computed tomography; PHE: perihematomal edema; ICH: intracerebral hemorrhage; T1WI: T1-weighted MRI; T2WI: T2-weighted MRI; FLAIR: fluid-attenuated inversion recovery; DWI: diffusion-weighted imaging; ADC: apparent diffusion coefficient.

The BBB consists of a continuous layer of endothelial cells joined by tight junctions [76]. Maintaining the normal function of brain capillary endothelial cells is essential to preserve the integrity of the BBB [76]. After acute ICH, ion channels and transporters in brain capillary endothelial cells undergo pre- and posttranscriptional changes [15]. These changes lead to abnormal ion transport and abnormal osmotic pressure, which ultimately facilitate the formation of PHE [15]. Progressive endothelial dysfunction further destroys the BBB and promotes the formation of vasogenic edema and potentially rebleeding [15]. According to the function of brain capillary endothelial cells, cerebral edema can be classified as ionic and vasogenic edema after ICH [25]. These two types of cerebral edema are continuous pathologic changes in the hemorrhagic brain [25]. Furthermore, the time phase of these two types of cerebral edema overlaps with cerebral edema induced by clot retraction, thrombin, and RBC degradation products [14, 25]. The classification and imaging characteristics of PHE are summarized in Table 2 and Figure 1.

### 3.1. Ionic Edema

Ionic edema is a subtype of albumin-deficient extracellular edema and has been defined independently of cytotoxic edema [25, 77]. It has been observed in the transition between cytotoxic and vasogenic edema after ischemic stroke [25, 77]. Cytotoxic edema is the result of failure of the ionic pump or the activation of selected ion channels induced primarily by energy metabolic dysfunction in neurons or astrocytes [78, 79]. Ionic edema refers to the net flow of transcapillary water from the capillary lumen to the interstitium of the brain through the endothelial cells of the BBB [12, 78]. That is, cytotoxic edema should be classified as intracellular edema, whereas ionic edema should be classified as extracellular or interstitial edema. Mechanism-wise, the dynamics of ionic edema is governed only by the osmotic term in the Starling principle [14, 22]. In ischemic stroke-induced ionic edema, the potential energy in the transendothelial Na^+^ gradient generated by cytotoxic edema drives the extravasation of electrolytes from blood vessels [14, 22]. First, Na^+^ is transported by endothelial cells to the brain parenchyma along the concentration gradient generated by cytotoxic edema, producing a nonzero osmotic driving force. Then, it drives chloride ions and water from blood vessels to the brain parenchyma to maintain electrical and osmotic neutrality, forming ionic edema [14, 22]. In other words, the ion influx of ionic edema is mediated by the primary and secondary active transport of Na^+^, Cl^−^, and water. The sulfonylurea receptor 1-transient receptor potential melastatin 4 (Sur1-Trpm4), the Na^+^-K^+^-2Cl^−^ cotransporter protein-1 (NKCC1), AQP4, the Na^+^-H^+^ exchanger, and the Na^+^-Ca^2+^ exchanger drive this process [79]. Because the pathogenesis of ICH is different from that of ischemic stroke, the mechanism of ionic edema after ICH should also be characterized. Cytotoxic edema has been shown to appear rapidly (in minutes) after interruption of cerebral blood flow in brain ischemia [25, 77]. However, studies on whether there is an “ischemic penumbra” in the perihematomal area are inconclusive [80, 81]. Therefore, it is also unknown whether cytotoxic edema will appear in the hyperacute phase of ICH. Except for the interstitial osmotic force established by clot retraction and serum protein extrusion from the hematoma, the role of the hematoma mass effect-induced reduction in blood flow and energy metabolism (including mitochondrial dysfunction and Na-ATP pump failure) may also warrant further exploration of ionic edema after ICH.

The time phase and imaging characteristics of ionic edema after ICH have not been clinically studied. However, both cytotoxic and ionic edema are triggered by the influx of fluid and Na^+^ [25]. Sur1-Trpm4, NKCC1, AQP4, the Na^+^-H^+^ exchanger, and the Na^+^-Ca^2+^ exchanger also drive the formation of cytotoxic edema [79]. Therefore, it is speculated that the imaging characteristics of PHE in patients may be similar to those of cytotoxic edema within 1-3 days [14, 27]. However, as previously illustrated, ionic edema is also characterized by extravasation of ions and accumulation of extracellular fluid (similar to interstitial edema) [12, 78]. Therefore, theoretically, the imaging characteristics of ionic edema in the perihematomal area can also resemble those of interstitial edema.

X-ray attenuation of brain tissue is directly correlated with its water content [82]. Therefore, CT can monitor the water content of brain tissue [82]. Cytotoxic edema and ionic edema present as brain tissue hypoattenuation seen on a plain computerized axial tomography (CT) scan [83]. Brain tissue with cerebral blood flow below 10 mL/100 g × min leads to ionic edema, which is accompanied by a decrease in Hounsfield units (HU) [82, 84]. Changes in CT image will be visible when the CT window is 40 to 60 HU [82, 84]. Furthermore, the Alberta Stroke Program Early CT Score (ASPECTS) was thought to be helpful for the identification and quantification of ionic edema [82, 84]. However, all the above studies did not consider distinguishing ionic edema from cytotoxic edema on CT images. Furthermore, the sensitivity of CT to ionic brain edema has not yet been determined because a reference standard is lacking under clinical conditions. Regarding MRI detection, both cytotoxic edema and interstitial edema show an increase in T2-weighted MRI (T2WI) and fluid-attenuated inversion recovery (FLAIR) images and a decrease in T1-weighted MRI (T1WI) [85–88]. Specifically, cytotoxic edema shows a reduction in signal on ADC maps but an increase in DWI. On the contrary, interstitial edema presents a nonhigh signal on DWI and a mild high signal on ADC maps [87–89]. However, the characteristics of the MRI image of ionic edema in the perihematomal area have not yet been disclosed.

Although a definitive conclusion has not been reached, studies have been conducted on the imaging characteristics of PHE in different stages of ICH. A study with MRI revealed that cerebral edema in the perihematomal tissues with imaging characteristics similar to cytotoxic edema appeared in at least half or more of the 21 patients enrolled within the first 24 hours. It also showed that the ADC values decreased until day 3 and were significantly reversed from days 3 through 7 [90]. An additional study indicated that the mean regional ADC level after ICH was lower at seven days than at 48 hours in perihematomal areas, suggesting that the transition from acute to subacute phase is characterized by a progressive resolution of perihematomal vasogenic edema associated with an increase in ADC values [91]. However, some studies illustrated that PHE might be cytotoxic or vasogenic in the hyperacute phase of ICH; it can be vasogenic or vasogenic accompanied by cytotoxicity in the acute phase of ICH, while it is always vasogenic in the subacute phase in patients who underwent an MRI scan [28–31, 92, 93]. Recently, a study suggested that ADC values in brain tissues with ionic edema increased in humans who underwent normobaric hypoxia [94]. Therefore, it is critical to further study whether the imaging features of ionic edema are similar to cytotoxic edema or interstitial edema in the perihematomal tissues after ICH. The chances are high that the imaging features of ionic edema resemble those of interstitial edema. It is also essential to clarify further whether there is an “ischemic penumbra” and whether cytotoxic edema can appear in the perihematomal tissues after acute ICH, as there has been evidence that indicates that cerebral edema in perihematomal tissues with imaging characteristics of cytotoxic edema prefers to appear in patients with larger hematomas [95].

Furthermore, evidence has indicated that a large hematoma can compress perihematomal tissues and reduce blood flow in this area after acute ICH [30, 96, 97]. The ongoing development of noninvasive imaging techniques should focus on their ability to identify and quantify ionic edema. Histological and imaging techniques should also explain the reasons for the appearance of imaging characteristics such as cytotoxic edema in perihematomal tissues after acute ICH.

### 3.2. Vasogenic Edema

Vasogenic edema is characterized by extravasation and extracellular accumulation of fluid in the cerebral parenchyma caused by disruption of the BBB [98]. The critical difference between it and ionic edema is that the former contains extravasated plasma proteins, while the latter does not [98]. In vasogenic edema, penetration holes are formed in the endothelial cells. They allow water molecules and plasma proteins to flow into the brain parenchyma, while capillaries maintain their structural integrity and prevent extravasation of RBCs [98]. Hydrostatic and osmotic pressure also play an essential role in the formation of vasogenic edema [98]. For example, systemic blood pressure must be kept high enough to maintain brain perfusion, but, in excess, it will promote the formation of edema [15]. In addition, the ICP must be kept low enough to maintain tissue perfusion, but high enough to counteract edema influx [15]. Optimization of these parameters is a multifactorial problem.

Animal research has revealed that vascular permeability peaked on day three after ICH [53]. PHE also peaked on day three and persisted for up to 7 days in experimental ICH models [14, 53]. However, the volume of PHE measured by MRI in patients increased more rapidly in the first two days after the onset of the symptoms and peaked toward the end of the second week [21]. As previously illustrated, the characteristics of vasogenic edema are similar to those of cytotoxic edema on CT, T1WI, T2WI, and FLAIR images [85, 86]. However, unlike the latter, vasogenic edema presents a normal to low signal on DWI and a high signal on ADC maps, which may resemble interstitial edema in perihematomal tissue after ICH [89].

The pathological process of PHE after ICH is complicated and has not been elucidated. Suppose the imaging features of ionic edema are finally verified to be similar to those of interstitial edema. In that case, it will be plausible to determine the time phase of ionic or vasogenic edema in perihematomal tissues with CT or normal MRI series [77]. Additionally, PHE also manifests itself as perihematomal hypodensity on CT images that can be difficult to distinguish from normal tissue and other entities that are also hypodense (e.g., infarction). As a result, the neuroimaging changes seen on MRI that depict the evolution of PHE warrant further observation. It is also critical to explore and quantify the cellular and molecular events underlying the multimodal imaging changes of PHE in different phases of ICH.

## 4. Quantitative Methods for PHE after ICH

As illustrated previously, PHE has been used as a promising surrogate marker for secondary brain injury after ICH [99]. However, the clinical predictive value of PHE for long-term functional outcomes in ICH patients has not been well established [100]. In addition, a proven PHE detection and quantification method can benefit the selection of treatment regimens and the evaluation of the prognosis. The following sections will introduce recent advances in detection and quantification methods to evaluate PHE in preclinical and clinical studies. The usages and limitations of different protocols for the quantification of PHE are summarized in Table 3.

### 4.1. Preclinical Studies

#### 4.1.1. Brain Water Content

The water content of the brain is widely used as a parameter to evaluate the severity of brain injury in animal ICH models. The percentage of brain water content is calculated as [(wet weight–dry weight)/wet weight] × 100% [9, 101]. It can be used to approximate changes in brain water in different regions of the brain after ICH in animals, including the ipsilateral and contralateral hemispheres and the cerebellum [9, 101]. However, the water content of the brain does not accurately reflect PHE due to the difficulty of directly separating perihematomal tissue from normal brain tissue.

#### 4.1.2. Brain Swelling

Brain swelling is one of the most widely used methods for quantifying the extent of brain edema in animals. It is calculated according to the following formula: [(ipsilateral hemisphere volume − contralateral hemisphere volume)/contralateral hemisphere volume] × 100% [9, 34, 101]. However, it cannot detect PHE directly in animals due to issues such as brain water content measurements. Furthermore, variation in the volume of the hematoma can also influence the precision of this protocol.

#### 4.1.3. MRI Image

As illustrated previously, the formation of PHE involves an increase in the permeability of the BBB. Although Evans blue extravasation has been widely used to assess BBB integrity, its use is limited, especially when the BBB remains intact in the hyperacute phase of ICH [102]. Therefore, the researchers also explored the quantitative value of different neuroimagings for PHE. Although Gd-based imaging (MRI and X-ray fluorescence) may help overcome the limitations of Evans blue staining [103], these methods also cannot be used to quantify the volume of PHE in animals. Instead, a 3.0 T or higher field MRI routine scan may represent an exciting means to directly measure the volume of brain edema or brain swelling in animals with acute ICH [53, 104–106]. Compared to other methods discussed above, the quantification of PHE volume in animals with MRI (T2-FLAIR) images should be more intuitive and accurate [107]. However, because of the limited access to high-field magnetic resonance imaging for small animals, its application in preclinical research remains limited. Although our previous findings have shown that the maximum volume of PHE in T2-FLAIR and ADC images (on day 3 after ICH) is consistent with the peak time point of brain water content and Evans blue concentration (ipsilateral/contralateral hemisphere) in ICH mice [53], studies on PHE volume detection and quantification with MRI in ICH animals are currently rare. Therefore, research should be carried out to evaluate the efficacy of the intervention by incorporating PHE quantified with MRI as an early endpoint indicator in ICH animals. Furthermore, the causal relationship between PHE detected with MRI and the long-term functional outcome may warrant further exploration in ICH animals to facilitate a potential translational possibility.

### 4.2. Clinical Studies

#### 4.2.1. CT and MRI

CT and MRI are the routine diagnostic methods used to quantify PHE in patients with ICH. PHE is defined as the hypodensity area adjacent to the hematoma on a CT image [108]. It exhibits characteristic hyperintensity on MRI (T2-FLAIR) in the perihematomal area [21, 108].

The PHE on CT images can be delineated by highlighting the hematoma and the edge of the hypodense area surrounding the hematoma on axial slices using the software's region of interest (ROI, cm^2^) module with the semiautomated edge detection tool or manually [108–112]. Similarly, the volume of PHE on CT images can also be calculated manually or with some semiautomatic/automatic computer-based methods [113]. Manual PHE CT volumetry is performed by subtracting the ICH volume measured separately from the total lesion volume calculated by multiplying the traced area (ROI, cm^2^) by the thickness of the slice [113]. However, the low reliability of manual CT volumetry evaluation for PHE may limit its clinical use [112–114]. Alternatively, threshold-based semiautomatic/automatic assessment is currently the method of choice in CT-based PHE volumetric assessment. However, the choice of suitable thresholds remains challenging because a validation group must be established to obtain the value of the identified HU thresholds [113]. To reduce the effort and time of physicians to segment and calculate PHE volumes in primary ICH patients, a study group recently proposed a deep learning model for automatic segmentation and volume analysis of PHE volume without setting thresholds [114]. As previously illustrated, CT images can be easily acquired. Therefore, the predictive value of the PHE volume detected with these deep learning models on the prognosis and treatment decisions of primary ICH patients may warrant further exploration. Regarding MRI, an investigation has indicated that the results of PHE measurements based on CT and MRI were consistent [108].

Furthermore, there is also evidence that the MRI method for the volumetric assessment of PHE is superior to CT in the delineation of PHE due to the high contrast between the edematous regions and the surrounding unaffected brain tissue clinically [113]. Therefore, MRI may represent the gold standard for detecting and quantifying PHE in humans. The PHE volumes in the MRI images (T2-FLAIR) can be calculated by multiplying the traced area (ROI, cm^2^) delineated with the semiautomated edge detection tool by the thickness of the slice [108, 115].

Based on CT or MRI images, there are a variety of established PHE evaluation parameters, including absolute PHE, relative PHE (rPHE), edema extension distance (EED), and PHE growth rate [21, 111, 115]. Absolute PHE refers to the sum of pixels surrounding the hematoma. Relative PHE (rPHE) refers to the ratio of the absolute PHE volume to the initial hematoma volume (rPHE = absolute PHE/initial hematoma volume) [109–111]. Studies on absolute PHE have indicated that more extensive hemorrhages lead to a larger volume of PHE [21, 116]. However, rPHE showed an inverse correlation with the initial volume of ICH, where a small ICH produced a volume of edema relatively more prominent than a large ICH [111]. To minimize the confounders caused by the volume of ICH, a recent study demonstrated that the edema extension distance (EED) could provide a better surrogate parameter in an early phase proof of concept clinical trial testing anti-inflammatory treatments [111]. EED is defined as the average thickness of PHE outside the hematoma boundary based on the hypothesis that the inflammatory response would extend a consistent mean linear distance-like edema from the hematoma boundary after ICH [111]. Furthermore, EED growth, calculated at different time points, can be used to detect PHE dynamic changes after ICH [115]. Although the use of EED as the primary outcome measure of edema can lead to reduced sample size requirements to evaluate the effects of variable treatment, the researchers suggested that additional confirmation is required in further analyses [115].

Furthermore, although EED growth at different time points was consistent with the progression of PHE in other human studies, the irregular shape of the hematoma may limit its use since it can only reflect the thickness of the edema exactly when the hematoma and edema are fully ellipsoid. As for other image markers, research has indicated that PHE's growth rate, known as the change in PHE volume per unit of time, may be a more robust parameter in ICH studies and could benefit from evaluating the mass effect beyond hematomas [109]. Additional large-scale studies may be necessary to justify the predictive value of the PHE growth rate on prognosis after ICH clinically.

Currently, the notion of whether PHE is an independent predictor of neurologic severity in human ICH has not been well established [109]. This critical knowledge gap severely impedes the translation of new therapies into clinics. Therefore, it is necessary to further evaluate and compare the predictive value of various image markers on neurologic outcomes in patients with acute ICH.

#### 4.2.2. Other Methods to Monitor Brain Edema or PHE

Although the midline shift on CT or MRI images may also clinically reflect brain swelling, the considerable variation in hematoma volume in patients with ICH limits its use [117]. Furthermore, it cannot accurately quantify the PHE volume. The main approaches, in addition to CT and MRI, for monitoring brain edema or PHE include the following: physical examination, indirect estimation of ICP (fundoscopy, displacement of the tympanic membrane, skull elasticity, and optic nerve sheath ultrasound), evaluation of cerebral blood flow (transcranial Doppler and Doppler of the ophthalmic artery), metabolic changes (near-infrared spectroscopy), and neurophysiological studies (electrical impedance tomography, electroencephalogram, visual evoked potential, and otoacoustic emission) [118]. Despite recent technological advances in various noninvasive techniques to monitor ICP, the current noninvasive standard does not replace invasive ones [119]. Since the principles of these noninvasive measurement methods are not sufficient to detect or monitor brain edema and could not directly reflect PHE, we do not illustrate their details and usage here.

## 5. Factors That May Impact PHE

According to the mechanisms of PHE formation discussed previously in The Time Phase and Pathophysiology, the evolution of PHE may be correlated with clot retraction, abnormalities in electrolytes and water transportation, activation of thrombin in the coagulation cascade, and toxicity of RBC degradation products in a different phase of acute ICH [14, 22]. Similar to the abnormality in electrolytes and water transportation seen in the DWI and ADC images, research has revealed that the appearance of hematomas in the T2∗-weighted images is heterogeneous with a non-hypointense core that may reflect hemolysis [58]. To investigate the variables that affect PHE, a clinical study determined the prevalence of early hemolysis in patients with ICH using MRI and attempted to indirectly identify the relationship between early hemolysis and perihematomal edema [59]. Data suggest a linear correlation between non-hypointense T2∗ lesions and the volume of perihematomal edema between days 1 and 14 [59]. Consistent with the results on the formation of PHE caused by intracerebral infusion of hemoglobin or erythrocyte lysate, early hemolysis in the hematoma occurred in humans and contributed to the development of perihematomal edema [59, 120]. However, recent advances in other easily accessible post-ICH variables need further exploration and will be reviewed here.

### 5.1. Preclinical Studies

Studies have revealed that, although rare, both age and gender influenced the severity of brain edema after ICH in mice [121, 122]. Aged male animals have more severe brain swelling [121, 122]. However, although there is evidence that therapeutic hypothermia may inhibit brain edema formation in animals with ICH, the significance of increases in temperature after ICH is currently unclear [123, 124]. However, it is unknown how genetics influences PHE in animals with acute ICH.

Furthermore, animal variables that influence PHE formation have not been studied compared to ICH patients. Therefore, more studies should fully consider the factors that can affect PHE formation in animals. Furthermore, it is essential to evaluate the efficacy of the cerebral venous drainage system and the pseudolymphatic system of the brain in PHE. Although almost no studies have explored the relationship between the cerebral drainage venous system and PHE in animals, there have been studies on the influence of the cerebral drainage venous system on PHE in patients with ICH [125–127]. Blocking of brain lymphatic drainage can exacerbate brain edema, neuroinflammation, and neuronal death and cause neurologic deficits in animals with acute ICH [128, 129]. Furthermore, the brain lymphatic drainage system is also involved in clearing brain waste from blood after ICH in animals [130]. Therefore, further exploration of the influence of the cerebral drainage venous system and brain lymphatic drainage system on PHE in animals may help identify therapeutic strategies for ICH.

### 5.2. Clinical Studies

A better understanding of the factors that influence the formation of PHE may be helpful in the development of potential treatment methods [131]. Unlike animal studies, numerous clinical studies have identified variables that may impact the evolution of PHE after ICH. Here, we summarize the variables that can aggravate PHE in patients in Table 4.

#### 5.2.1. Baseline and Clinical Variables

Variations in specific genotypes have been observed in patients with ICH [131–134]. Genetic characteristics of the apolipoprotein E (APOE), AQP4, tissue inhibitor of metalloproteinases 2 (TIMP-2), and haptoglobin phenotypes have been shown to correlate with early changes in PHE volume [131–134]. In particular, a study indicated that in patients with a similar volume of hematoma, the midline shift probably induced by increased cerebral edema was more prominent in patients with at least 1 APOE4 allele (APOE4^+^) than in patients with APOE4^−^ after acute ICH [132]. Additional studies on the AQP4 and TIMP-2 genes revealed that the variant of AQP4 in the rs1054827 and GC genotype at the position TIMP-2-418 (rs8179090) promotes the formation of PHE [133, 134]. However, studies on the impact of haptoglobin phenotypes on PHE are currently inconsistent. A study with 166 patients indicated that the Hp 1-1 haptoglobin phenotype, but not the Hp 2-1 or Hp 2-2 haptoglobin phenotypes, was associated with increased progression of PHE within the first 96 hours [131]. On the contrary, another study with 731 patients found that the haptoglobin phenotype did not influence the volume of PHE, the volume of ICH, or the functional outcomes, while the HP2-1 genotype could be associated with a lower mortality at 6 months [135]. It will be necessary to explore further the mechanisms underlying the association between these genotype variations and the evolution of PHE after ICH.

Regarding other demographic or clinical variables, EED studies revealed that PHE tended to be more severe inpatients of males, older age, a higher score on the National Institutes of Health Stroke Scale (NIHSS), a lower Glasgow Coma Scale score, a larger volume of ICH, larger initial EED, irregularly shaped hematoma, black hole sign, or higher glucose concentration [111, 115, 116, 136, 137]. Meanwhile, data from the Intensive Blood Pressure Reduction in Acute Cerebral Haemorrhage Trial (INTERACT) indicated that a history of hypertension, baseline hematoma volume, body temperature, and time from the onset of symptoms to CT scan were independently associated with a relative increase in PHE in 207 patients with acute ICH [138]. In particular, many studies implied that a large volume of ICH was correlated with the severity and progression of PHE [115, 116, 138, 139]. Probably due to the ambiguous effects of fever on ICH outcomes, the effects of therapeutic hypothermia on brain edema are currently heterogeneous in patients with ICH [123, 140, 141]. In addition to the demographic and imaging variables discussed above, other clinical variables may also influence the evolution of PHE after ICH. For example, a study showed that prehospital use of sulfonylureas predicted lower PHE volumes and lower rPHE on admission in 317 patients with diabetes mellitus and primary ICH [142].

Furthermore, another retrospective case-control study that included 9 patients with basal ganglia hemorrhage who received sulfonylurea pretreatment and 18 matched controls obtained similar results [143]. As previously illustrated in The Classification and Imaging Characteristics of PHE, systemic blood pressure (SBP) can promote the formation of edema after ICH. There were also additional studies on the influence of blood pressure on PHE. Although no decrease in the rPHE growth rate was observed at 24 hours in the Antihypertensive Treatment of Acute Cerebral Hemorrhage-2 (ATACH-2) trial, an inverse correlation was found between the SBP at admission and absolute PHE at 72 hours in the INTERACT trial [144–146]. Moreover, impaired blood pressure regulation measured by baroreflex sensitivity could also be an independent predictor of rPHE in patients with ICH [139]. Additional studies on demographic or clinical variables may help predict and treat PHE.

#### 5.2.2. Hematological Characteristics

Laboratory variables may influence the evolution of PHE. Some studies have observed the influence of hematologic characteristics on the severity or progression of PHE. An investigation revealed that the higher platelet count in the periphery was significantly correlated with the volume of PHE within the first 24 hours after ICH in a multiple regression analysis of 80 patients [147]. Similarly, a higher neutrophil-lymphocyte ratio was also found to represent a systemic inflammatory response at admission and was independently associated with PHE growth [117, 148]. Regarding variables in the coagulation and fibrinolysis system, evidence revealed that higher admission hematocrit levels and a longer partial thromboplastin time were associated with more significant delays in peak PHE and higher peak rPHE [21]. Research on warfarin-associated ICH also indicated that prior warfarin use was associated with a lower volume of rPHE or EED at admission [99, 149]. It is critical to further verify the influence of the prior use of antiplatelet or anticoagulant drugs on the growth of hemorrhage and the expansion of PHE. Furthermore, studies on the predictive value of more easily acquired serum biomarkers for PHE are needed, since other studies have found that acute serum levels of IL-6, soluble CD163, MMP-3, and MMP-8 were sensitive biomarkers to identify patients at risk of expansion of PHE [150–153].

#### 5.2.3. Cerebral Venous Drainage System

The cerebral venous drainage system plays a vital role in maintaining cerebral blood flow and physiological function of the brain [154]. It can be divided into superficial and deep venous systems attached to the internal jugular veins [154]. The harmony between arterial blood flow and venous output is crucial for brain homeostasis. There are many superficial veins, but the three most prominent are the superficial middle cerebral vein (SMCV), the Trolard vein (VOT), and the Labbe vein (VOL), while the internal cerebral veins (ICV), the Rosenthal basal veins (BVR), and the Galen veins are important constituents of deep venous systems [155]. The filling status of the cerebral veins can be visualized with a specific series of CT or MRIs such as CT perfusion and magnetic resonance venogram in humans [125].

Venous stenosis and outflow obstruction are present in the injured brain, probably induced by contraction of the pericyte and deposits of erythrocytes, leukocytes, platelets, and fibrin in the lumina of the vessel [154, 156]. Furthermore, damage to the cerebral venous drainage system has also been attributed to hypoperfusion and venous stenosis caused by the compressive effect of ICP [125–127]. Insufficient cerebral venous drainage has been observed in the hemorrhagic brain. Evidence indicated that approximately 1/3 of patients with ICH had absent ipsilateral venous filling in at least 1 of the 5 superficial or deep ipsilateral veins (SMCV, VOT, VOL, BVR, and ICV) around six hours after the appearance of ICH [125]. In contrast, insufficient cerebral venous drainage can also influence the severity of brain injury by promoting the formation of PHE. It has been revealed that there is a strong correlation between low perfusion of the corresponding drainage area of the cerebral vein and high rPHE 24 hours after the onset of ICH [125]. As illustrated previously, fluids from the superficial and deep venous systems influx into the internal jugular veins [155]. A study revealed that the volume of rPHE was significantly higher in ICH patients with jugular vein reflux (JVR) than in JVR-negative patients, further suggesting that damage to the cerebral venous drainage system promotes the development of PHE [157]. Cerebral venous outflow volume (CVFV) can also affect the formation of brain edema in ICH. For example, multivariate analysis showed that CVFV was an independent factor of late PHE (at 12 ± 3 days) after ICH [158].

The above findings suggest that there may be insufficient cerebral venous drainage in patients with ICH. Furthermore, they are associated with the early development of rPHE, which may be an effective imaging indicator to predict the risk of expansion and a potential therapeutic target in patients with ICH.

#### 5.2.4. Brain Lymphatic Drainage System

The brain lymphatic drainage system, including the glymphatic system and meningeal lymphatic vessels, may also provide a channel for fluid influx or efflux into the brain parenchyma after stroke [128, 159, 160]. The glymphatic system comprises the drainage pathways of perivascular and cerebrospinal fluid, which allow the CSF to flow into the brain parenchyma through penetrating arterial perivascular spaces and are facilitated by AQP4 water channels expressed in astrocyte processes [159, 161–165]. It also has a route that drains interstitial fluid (ISF) from the brain parenchyma to the cervical lymph nodes (CLN) or the venous bed [160, 166, 167]. However, the meningeal lymphatic vessels near the venous sinus in vertebrate brains may be a unidirectional drainage route for cerebrospinal fluid and ISF into adjacent lymph nodes or the blood stream [160, 168]. Brain edema is an abnormal accumulation of excess water in the brain parenchyma that results in swelling of brain tissue. Therefore, it is crucial to investigate the relationship between the brain lymphatic drainage system and edema after acute stroke.

Glymphatic and meningeal lymphatic dysfunction of the brain has been observed in patients with ischemic stroke [169–171]. This abnormality may promote the formation of brain edema in some neurological disorders, including ischemic stroke and subarachnoid hemorrhage. Mechanism-wise, impairment of the perivascular pathway and dislocation of AQP4 in the glymphatic system are involved in the formation of edema after ischemic stroke or subarachnoid hemorrhage [159]. Furthermore, increased ICP can also influence edema formation by regulating meningeal lymphatic drainage after ischemic stroke or traumatic brain injury [159, 165, 172]. Additional studies on the role of the brain lymphatic drainage system in the clearance of molecular waste, the maintenance of homeostasis, and immune surveillance suggest that the dysfunction of the brain lymphatic drainage system may have the ability to aggravate brain edema by promoting an inflammatory response in the injured brain [159, 165, 173].

**Figure 2:**
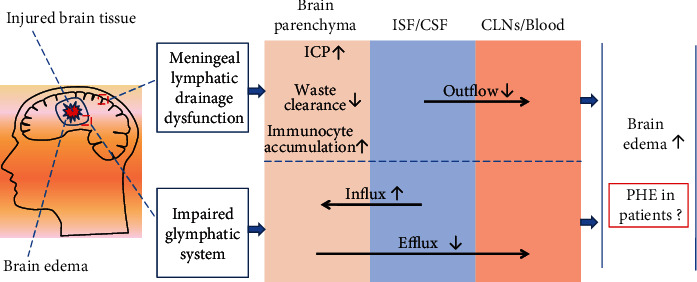
Impairment in brain lymphatic drainage and the formation of cerebral edema. The meningeal lymphatic system constitutes the brain lymphatic drainage system in the dorsal part of the skull and the glymphatic system (a glia-dependent system of the perivascular space) present in the brain parenchyma. The meningeal lymphatics are involved in maintaining homeostasis and immune surveillance in the brain. The glymphatic system provides a pathway to remove interstitial solutes and wastes in the brain parenchyma. It is also a bidirectional exchange pathway between ISF and CSF. However, the meningeal lymphatic system may only function as a drainage pathway. The brain lymphatic drainage system is a crucial drainage route for ISF/CSF into the cervical lymph nodes (CLNs) or peripheral blood. Brain injury may alter the drainage function of the meningeal lymphatic system and glymphatic system and subsequently aggravate brain edema after ischemic stroke, subarachnoid hemorrhage, TBI, etc. High ICP may reduce the flow of the lymphatic system from the ISF/CSF to the CLN or the venous sinus. Impairment in the glia-dependent system of the perivascular space, especially dislocation of AQP4 in the endfeet of the astrocyte of the glymphatic system, can lead to an increase in ISF/CSF influx to the brain parenchyma with a decrease in efflux from the brain parenchyma to ISF/CSF or CLN/blood. Reduction in the function of the lymphatic and glymphatic systems can also lead to a decrease in waste clearance and an increase in immunocyte accumulation in the injured brain. Although animal studies have indicated that brain lymphatic drainage dysfunction may facilitate the formation of brain edema after ICH, no studies have explored its relationship in patients with ICH. Abbreviations: CSF: cerebrospinal fluid; ISF: interstitial fluid; CLNs: cervical lymph nodes; TBI: traumatic brain injury; ICP: intracranial pressure; AQP4: aquaporin 4; ICH: intracerebral hemorrhage.

Regarding ICH, although previous studies have illustrated that abnormalities in the brain lymphatic drainage system can promote the formation of brain edema in animals [128, 129], no research has provided information on changes in the brain lymphatic drainage system and its relationship with PHE in patients with ICH [161–164]. Relevant future studies will provide information on the mechanism of post-ICH PHE formation. Additional studies can help to find an imaging indicator to predict the severity of PHE in patients with ICH. The influence of the brain lymphatic drainage system on brain edema after brain injury is summarized in Figure 2.

## 6. PHE and Functional Outcomes

The primary objective of most studies on the mechanisms, diagnosis, and quantification of PHE is to seek methods to alleviate the severity of brain injury and promote functional recovery after ICH. Therefore, it is critical to evaluate the relationship between PHE severity and the prognosis of ICH. Suppose that we establish a positive correlation between them. In that case, it will be reasonable to further identify whether PHE can be used as a potential therapeutic target or an endpoint indicator to evaluate the efficacy of different interventions. We discuss recent advances in the relationship between PHE severity and ICH prognosis.

### 6.1. Preclinical Studies

Evidence has indicated that the appearance of brain edema and increased ICP could lead to an increase in the apoptosis of brain cells, which suggests that PHE may represent a promising surrogate marker for the efficacy of different interventions in the severity of early brain injury in ICH animals [13, 108, 174]. However, few studies have explored the causal relationship between PHE and the total volume of the lesion or the functional results of ICH animals [53]. Due to limited access to equipment, MRI is not commonly used to quantify PHE in ICH animals. Accurate and reliable quantification of PHE will be crucial for future ICH animal studies that use PHE as a surrogate indicator of the outcome.

### 6.2. Clinical Studies

Neuroimages can be easily acquired in patients with acute ICH. Clinical studies have evaluated the predictive value of PHE on functional outcomes with neuroimages after acute ICH (Table 5). Regarding absolute PHE volume, a study with 292 patients showed that the maximum PHE volume could represent an independent predictor of functional outcome on day 90 after ICH [100]. Furthermore, two clinical studies revealed that the high absolute volume of PHE within 12 h or on days 1-12 after symptom onset was correlated with a negative functional effect, representing a possible treatment target [20, 175]. Additional studies in specific patient groups (e.g., prehospital use of nonoral anticoagulants or patients with chronic kidney disease) also revealed that an absolute increase in PHE within 12 or 48 h after ictus was an independent predictor of poor outcome after ICH [99, 176]. It seems that the variation in time points in the acute or subacute phase does not impact the predictive value of absolute PHE on functional outcome after ICH. However, a single-center prospective study with 133 patients suggested that the effect of absolute PHE volume within 24 h after symptom onset on functional outcome depends on the smaller size of the hematoma [177]. It was found that absolute critical PHE volume is a significant predictor of poor outcome only in patients with ICH volumes < 30 mL, suggesting that hematoma volume may affect the relationship between absolute PHE and prognosis of ICH [177]. Furthermore, an additional study with 342 patients with a median total lesion volume of 48 mL also indicated that the volume of PHE on a diagnostic brain CT, taken within three days, was not independently associated with death or dependence one year after ICH [178]. Therefore, it may be logical to further verify the influence of the volume of the hematoma on the relationship between absolute PHE and the prognosis of ICH in stratified patients with small and large volumes of hematomas.

The growth of PHE measured by PHE expansion and EED was also used to evaluate the severity of brain edema after ICH in patients. Two clinical studies have found that an increase in absolute PHE volume from baseline to day 3 was correlated with a poor outcome 3 months after ICH [116, 179]. A study with 115 patients also found that the PHE expansion rate from baseline to 72 hours was associated with poor functional outcomes after deep ICH [180]. On the contrary, the PHE expansion rate from baseline to 24 hours was associated with mortality after deep and lobar ICH [180]. Furthermore, another study with 139 patients revealed that a faster expansion rate of PHE 24 hours after ICH was associated with a worsening outcome [109].

Similarly, a study with 596 patients showed that the absolute increase in PHE within 6 to 72 hours after the onset of ICH was associated with worse functional outcomes in patients with basal ganglia ICH and hematomas < 30 mL [181]. Regarding EED, a large study with 1,028 cases demonstrated that EED for 72 h is independently associated with deteriorating neurologic deficits at 90 days [136]. Another study that included 861 patients also found that the EED growth rate over 72 h was an independent risk factor for mortality in patients with ICH [115]. However, a study illustrated that the growth of edema volume from the first MRI on admission to 48 h was only correlated with a decrease in neurologic status at 48 h (81 vs. 43 mL, *P* = 0.03), but not with the functional outcome [21]. The median ICH volume of this study is 39 mL; thus, growth in PHE volume in patients with a larger hematoma volume can predict prognosis. Furthermore, although most of the above findings imply that the progression of secondary brain injury detected by PHE growth appears to be an effective indicator of the prognosis of ICH in patients, it is also essential to illustrate whether the location of the hematomas influences the predictive value of PHE growth on the prognosis of ICH clinically.

Regarding the relationship between rPHE and functional outcome, a study with 38 patients indicated that rPHE at 48-72 h after ictus independently predicted early neurologic deterioration [139]. Another study also revealed that absolute PHE and rPHE on day 3 were significantly associated with death or dependency 90 days after ICH [138]. However, other evidence proved that baseline rPHE within 3 h after ICH onset rather than 1 and 20 h later was associated with poor functional outcomes but did not predict mortality [182]. On the contrary, the volume of absolute edema predicted neither mortality nor functional outcome in this study [182]. However, another study indicated that an increase in absolute PHE between days 1 and 3, but not rPHE, was predictive of in-hospital mortality in patients with ICH [110].

Similarly, a meta-analysis of 21 studies also yielded controversial values of absolute PHE and rPHE as prognostic markers, suggesting that PHE measures and time points and results vary in previous studies [183]. An abnormal increase in rPHE in ICH patients with a small volume of hematoma may explain some of the above negative findings [111, 181]. Additionally, due to its inverse correlation with the volume of ICH, relative PHE may not be suitable for analysis considering the clinical impact of PHE [110]. Therefore, more studies are needed to compare rPHE values with absolute PHE or other measurements as prognostic markers after ICH.

In summary, although the relationship between PHE and the prognosis of ICH is currently controversial and inconsistent, absolute PHE and PHE growth seem to be more reliable for predicting the prognosis of ICH compared to rPHE. As illustrated in Quantitative Methods for PHE after ICH, MRI is more accurate than the CT image to detect PHE. Most studies on the relationship between PHE and ICH prognosis quantified PHE with CT but not MRI for the use of MRI is limited by time-consuming, expensive fees, and difficulty in movement of ICH patients. Furthermore, the quantification time point, size, and location can also influence the predictive value of PHE on the prognosis of ICH. Therefore, it is critical to further determine the appropriate time point for the quantification of PHE in a unified site such as the basal ganglia on MRI to predict the prognosis of patients with ICH. It is also essential to further evaluate the accuracy of CT for PHE quantification by comparing it with MRI, because CT images can be easily acquired. Therefore, the causal relationship between PHE severity and functional outcomes still warrants further exploration with rigorously designed studies.

## 7. Potential Therapeutic Targets for PHE after ICH

Despite recent attempts to discern the pathophysiology of ICH [17], evidence-based therapies for ICH are not yet available [3]. Current treatment is primarily supportive, and the outcomes of patients with ICH remain poor [3]. As illustrated previously, the growth of PHE may aggravate secondary brain injury and subsequently worsen neurologic deficits [13, 14]. Although the predictive value of PHE for the therapeutic values of different interventions in ICH still warrants further verification, studies have tried to explore whether brain edema, specifically PHE, could be targeted to promote functional recovery after acute ICH [12]. The following section will discuss potential targets to alleviate PHE.

### 7.1. Preclinical Studies

As the mechanism of PHE has been extensively investigated in animals, almost all preclinical studies focus on alleviating PHE by targeting factors that contribute to the formation of PHE after acute ICH [14, 22]. Here, we summarize the promising therapeutic approaches targeting PHE in animals (Table 6). Unfortunately, the use of high-field MRI is limited to small animals, and alternative reliable PHE quantification tools are lacking. Thus, studies of PHE are only broadly described.

#### 7.1.1. Dehydration Therapy

Research has revealed that mannitol, not furosemide, produces a dose-dependent increase in plasma osmolality and reduced brain water content in male Sprague-Dawley rats [184]. However, the combination of furosemide with mannitol resulted in a more significant increase in plasma osmolality than mannitol alone, with a more substantial decrease in brain water at 4 and 8 g/kg of mannitol [184]. Furthermore, research revealed that mannitol and normal hypertonic saline (HTS) increased plasma osmolarity 1 h after infusion (315 ± 4.2 and 310 ± 2.0 vs. 301 ± 1.5 mOsm/kg), reduced mortality at 48 h (82, 36, and 53%, respectively), and inhibited hemispheric swelling 48 h after ICH [185]. In addition to the dehydration mentioned previously, mannitol and HTS can also alleviate inflammatory responses by inhibiting the activation of microglia/macrophages and the infiltration of CD45^+^ cells into the perihematomal tissues of animals with ICH [185]. However, albumin also exhibits neuroprotective capacity by mitigating oxidative stress after acute ICH in rats [186], suggesting further exploration of the effects of these agents on PHE, especially the effects of mannitol and albumin warranted in both preclinical and clinical settings.

#### 7.1.2. Inhibition of Thrombin Activation and Clearance of RBC Hemolysates

Thrombin activation is involved in early PHE formation. Therefore, inhibition of thrombin activity can mitigate PHE or early brain injury after acute ICH [32, 37, 40]. Research has revealed that thrombin promotes the disruption of the BBB and aggravates brain edema through PAR (PAR-1, PAR-3, and PAR-4) and its downstream signaling after acute ICH [37]. There has yet to be a study indicating that direct oral anticoagulants (DOAC) limit thrombin toxicity in endothelial cells (of the BBB), likely by inhibiting PAR-1 activation [187]. Furthermore, evidence also indicated that 12-(3-adamantan-1-yl-ureido)-dodecanoic acid (AUDA), a selective soluble epoxide hydrolase inhibitor, reduced microglial activation *in vitro* and reduced MMP-9 activity and BBB disruption on day 1 after acute ICH *in vivo* [188]. These studies suggest that thrombin may represent a potential target for alleviating PHE after acute ICH.

The degradation products can cause brain edema after acute ICH [13]. However, research findings on the effects of iron chelation on brain edema are not consistent. A study in rats indicated that bipyridine, an iron chelator, does not reduce brain edema or improve outcome after ICH [189]. Additional research in mice and rats also indicated that deferoxamine (DFX), another iron chelator, does not reduce brain swelling or brain edema after ICH [190, 191]. However, other studies found that 5-[4-(2-hydroxyethyl) piperazine-1-ylmethyl]-quinoline-8-ol (VK-28) and 2,2′-dipyridyl, two brain-permeable iron chelators, reduced the volume of PHE and the neurologic deficit in animals with ICH [101, 192]. Furthermore, it has been observed that, compared to intraperitoneal injection of deferoxamine mesylate (DFO) alone, in situ intracerebral administration of DFO-loaded thermosensitive keratin hydrogels (TKG) effectively reduced iron deposition and nonheme iron content, inhibited the reactive oxygen species (ROS) production, and alleviated brain edema [69], suggesting that TKG conjugated with permeable iron chelators of the brain may have translational potential to treat iron-induced brain injury after ICH. Alternatively, a recent animal study also showed that inducers of NF-E2-related factor 2 (Nrf2) and peroxisome proliferator-activated receptor *γ* (PPAR*γ*) reduced BBB permeability and alleviated brain edema by accelerating hematoma clearance after acute ICH [71]. However, another study implied that IL-10 may enhance microglial phagocytosis for RBCs and reduce brain edema by increasing the expression of CD36 expression in the hemorrhagic brain of mice [57]. These findings indicate that the efficacy of inhibiting thrombin activation and the release of hemolysates of RBC in PHE warrants further preclinical ICH research.

#### 7.1.3. Inhibition or Modulation of the Inflammatory Response

After ICH, the hemolysate-induced inflammatory response contributes to PHE formation. Therefore, it may be feasible to reduce brain edema by inhibiting microglial/macrophage activation, the release of proinflammatory factors, and the chemotaxis of peripheral blood immunocytes [10, 193]. Toll-like receptor 4 (TLR-4) can initiate an inflammatory response after acute ICH [193, 194]. Studies have reported that the TLR-4 antagonist reduced brain edema and neurologic deficits after ICH [195, 196]. Regarding thrombin-associated inflammation, evidence shows that adenovirus-mediated overexpression of the IL-1 receptor antagonist attenuated brain edema formation after ICH, perhaps by reducing thrombin-induced brain inflammation [197]. Additional research on complement mediators revealed that blocking the C3a or C5a receptors also reduced the water content and alleviated neurologic deficits in hemorrhagic stroke [198, 199]. Furthermore, treatment with CD47 blocking antibodies accelerated hematoma clearance through microglia/macrophages and reduced ICH-induced brain swelling, loss of brain tissue, and neurologic deficits, suggesting that CD47 blockade has potential value in combination with surgical blood clot clearance [104].

Regarding immunomodulation, a previous study revealed that intraperitoneal administration of fingolimod (FTY720, an analog of the sphingosine 1-phosphate receptor (S1P) (S1PR), the first generation of S1PR modulators) significantly alleviated brain edema and improved functional recovery of ICH mice [200]. Furthermore, siponimod (BAF-312), a selective antagonist of S1P receptor types 1 and 5, also increased the survival rate, alleviated PHE, and relieved ICH-induced sensorimotor deficits [107, 201]. Mechanism-wise, additional research has indicated that the administration of CAY10444, an S1PR3 antagonist, led to reductions in brain edema volume, improvements in BBB integrity, and alleviation of behavioral deficits by inhibiting CCL2 and p38MAPK in rats with ICH [202]. Although the underlying mechanism has not been fully elucidated, S1PR may exert its neuroprotective effects by inducing the regulation of brain inflammation and then mitigating secondary brain injury after ICH [107, 203, 204]. Furthermore, studies on minocycline revealed that it also improved the consequences of ICH by preserving the integrity of the BBB and attenuating neurologic deficits by increasing the dickkopf-1 (DKK1)-Wnt1-*β*-catenin or the TGF-*β*-mediated MAPK signaling pathways in animals [36, 205]. Currently, immunomodulation has been suggested as a promising strategy for the treatment of ICH. The results of these studies encourage further exploration of immunomodulators and their related drugs to promote translation and alleviate PHE after ICH.

#### 7.1.4. Other Therapy

Although there are some studies that indicated that treatment with either glibenclamide or bumetanide does not lessen striatal edema or improve outcome following collagenase-induced ICH in rats, most recent studies have indicated that drugs used to treat or prevent cerebrovascular diseases, including simvastatin, rosiglitazone, pioglitazone, glibenclamide, and nicardipine, could alleviate brain edema after acute ICH in animals [206–213]. Furthermore, protecting and repairing injured vascular endothelial cells may represent a potential target to inhibit brain edema formation after ICH [214]. Research on adipose-derived mesenchymal stem cell (ADSC) transplantation revealed that it reduced brain edema by inhibiting inflammation and AQP4 protein expression after ICH [215]. Although further verification may be necessary, other research showed that repetitive transcranial magnetic stimulation (rTMS) reduced brain edema in ICH mice through the MAPK signaling pathway [216]. Furthermore, evidence also indicates that therapeutic hypothermia may promote behavioral recovery by alleviating BBB disruption and reducing cerebral edema in animals treated with ICH [140]. More animal studies must be conducted to search for alternative treatment methods for the alleviation of PHE.

Briefly, a large group of animal studies have tested the values of different interventions on brain edema or PHE after ICH. However, as previously illustrated at the beginning of this section, most of the animal studies discussed above evaluated PHE indirectly with histological methods, and only a few studies detected PHE directly with MRI images. The translational value of these animal studies still deserves further exploration. Furthermore, age, gender, and comorbidities (e.g., diabetes mellitus) have a profound impact on the outcome of ICH [121, 122]. The quality of animal reporting may also be influenced by sample size, inclusion and exclusion criteria, randomization, etc. [217, 218]. Based on the recommendations of the Initial Stroke Therapy Academic Industry Roundtable (STAIR) and the requirements of the ARRIVE (Animals in Research: Reporting In Vivo Experiments) Guidelines for Reporting Animal Research *in vivo* [217–219], more rigorous animal studies should be designed to further evaluate the values of different interventions in PHE by quantifying PHE with high-field MRI.

### 7.2. Clinical Studies

Clinical studies also attempted to alleviate neurologic deficits by limiting secondary brain injury after acute ICH. The efficacy of dehydration therapy, blood pressure control, hemostasis, immunotherapy, and surgical treatment has been tested in patients with acute ICH. We summarize recent advances in potential therapeutic methods to treat PHE after acute ICH (Table 7).

#### 7.2.1. Dehydration Therapy

In addition to a reduction in cerebral blood and cerebrospinal fluid volumes, shrinkage of the volume of neurons and astrocytes in areas far outside the site of injury, including the contralateral hemisphere, can provide further compensation to counteract ICP [220]. However, once these compensations fail, additional brain damage will occur. Dehydration therapy is used to reduce ICP for various brain disorders. Hyperosmolar agents, including hypertonic saline, mannitol, glycerin, fructose, and albumin, are often used to create an intravascular osmotic gradient for dehydration after acute ICH [221, 222]. However, there is insufficient evidence to recommend the routine or prophylactic use of hyperosmotic agents in ICH [3]. Hyperosmotic agents (mannitol or 3% normal saline) can be considered a temporizing measure to decrease ICP in patients with ICH with clinical signs of herniation before surgical intervention [3]. In addition, the efficacy of other dehydration agents, such as furosemide, glucocorticoid, and acetazolamide, has not been verified in the treatment of patients with ICH [3]. Furthermore, the use of corticosteroids to reduce ICP may cause harm without proven benefits and is therefore currently not recommended for clinical use [3, 221]. However, the low or modest strength of the original evidence discussed above may not reflect the true effect of the dehydration agents, and further research is still needed. Furthermore, there is also evidence that brain water content in the hemorrhagic hemisphere was not correlated with ICP in rats with ICH [223, 224]. Thus, it is also critical to further validate whether dehydration therapy can alleviate the severity of PHE and subsequently promote functional recovery in patients with acute ICH.

#### 7.2.2. Blood Pressure Control

It is not clear whether a decrease in blood pressure (BP) will mitigate the secondary brain injury from ICH. A recent study has shown that intensive BP control is associated with a decreased perihematomal edema expansion rate (PHER) at 24 hours in deep ICH, which in turn is associated with adverse outcomes in basal ganglia ICH but not in all deep ICH (e.g., thalamic ICH) [144]. Another randomized controlled trial study revealed that the volume of the hematoma at 24 hours, the volume of PHE at 72 hours, and the NIHSS scores at 30 and 90 days in patients receiving intensive antihypertensive treatment were lower than those of the placebo group, while the Barthel scores at 30 and 90 days were higher [225]. Furthermore, a study with 635 ICH patients illustrated that angiotensin-converting enzyme inhibitors and angiotensin II receptor blockers reduced mortality, volume of perihematomal edema, and prevalence of pneumonia in patients with hypertension [226]. A recent guideline showed that BP to a target of <140 mm Hg systolic does not worsen neurologic deficits compared to 180 mm Hg systolic; however, no clinical benefit has been established [3]. Furthermore, there is no adequate evidence to guide the choice of initial BP-lowering agents [3]. More research is needed to explore the efficacy of BP control in PHE and long-term neurologic recovery after ICH.

#### 7.2.3. Hemostasis and Iron Chelator

Hematoma expansion (HE) leads to adverse outcomes in patients with acute ICH [227, 228]. Previous studies have tried to evaluate the effects of hemostatic therapy on the expansion of hematoma and functional recovery after ICH [227]. Most studies revealed that hemostasis with vitamin K antagonists, fresh frozen plasma (FFP), activated factor VII (rFVIIa), prothrombin complex (PCC), tranexamic acid, and aminocaproic acid targeted in the hematoma did not improve outcomes [227, 229, 230]. However, almost no research has explored the effects of hemostasis on PHE after acute ICH. Furthermore, in a phase 2 multicenter, randomized, placebo-controlled, double-blind trial with 294 participants, the investigators found that treating ICH patients with deferoxamine mesylate cannot improve the chance of a good clinical outcome (mRS 0–2) on day 90 after the onset of symptoms [231]. By stratifying the 294 participants into those with a small, moderate, or large hematoma volume, the investigators of the same trial found that a greater proportion of patients treated with deferoxamine than placebo achieved a modified Rankin scale score 0-2 among patients with moderate hematoma volumes (10-30 mL) but not in patients with small (<10 mL) or large (>30 mL) hematoma volumes [232]. Like the results on iron chelation therapy discussed in preclinical studies in this section, these findings have important trial design and therapeutic implications for the evaluation of iron chelator efficiency in ICH in the future.

#### 7.2.4. Immunotherapies

In patients with minor to moderately deep primary supratentorial ICH, fingolimod treatment within 72 hours after the onset of the disease was safe, reduced PHE, attenuated neurologic deficits, and promoted recovery compared to the placebo group [233]. Another study revealed that intravenous injections of siponimod were well tolerated, with safety and pharmacodynamic profiles (e.g., absolute lymphocyte count) similar to those of oral siponimod in healthy subjects [234]. However, the results of a triple-blind placebo-controlled intracerebral hemorrhage trial (NCT03338998) are not yet available. As for other immunomodulators, studies with minocycline revealed that although the serum level of MMP-9 tended to be lower in minocycline-treated patients than in placebo-treated controls, intravenous injection of minocycline did not influence PHE or functional outcomes [235, 236]. These findings on the effects of minocycline in patients contradict the results in animals with ICH. However, these studies may have type 2 errors due to their small sample sizes. Additionally, false positives may also exist in the above analysis owing to the same issue (low statistical power from small sample sizes). Therefore, the efficacy of fingolimod or other immunomodulators in PHE warrants further investigation.

#### 7.2.5. Surgical Management

Removal of the hematoma reduces the compression effect of blood clotting and alleviates the toxicity of bleeding degradation products after ICH. Many clinical studies have evaluated the efficacy of surgical management in acute functional outcomes of ICH compared to conservative treatment. On the contrary, the clinical benefit of craniotomy and minimally invasive clot evacuation has not yet been established [3, 11]. Regarding brain edema after acute ICH, studies on the effects of surgical treatment are also currently controversial. The results of a previous clinical study showed that burr hole craniectomy could reduce brain edema in patients with hypertensive basal ganglia hemorrhage, most likely by alleviating end-product toxicity [237]. A clinical study on decompressive craniotomy and stereotactic aspiration also confirmed that surgical evacuation of the hematoma could significantly reduce the volume of the hematoma and PHE in patients with putamen hemorrhage than in the conservative treatment group [238]. However, another clinical study showed that decompressive craniectomy only reduced the midline shift, probably by improving the mass effect of the hematoma, while dramatically increasing the absolute volume of PHE compared to conventional treatment [239]. Currently, there is no conclusive evidence that craniotomy can mitigate the severity of brain injury by alleviating PHE formation in patients with ICH. Although the efficacy of minimally invasive clot evacuation with stereotactic or endoscopic aspiration with or without thrombolytic use is uncertain, some studies suggest that minimally invasive approaches are superior to craniotomy [4, 11, 240]. A study with 36 patients revealed that minimally invasive surgery (MIS) could alleviate PHE in ICH patients [241]. In another cohort study, MIS combined with the recombinant human tissue-type plasminogen activator (rtPA) inhibited early or delayed PHE formation and reduced mortality after ICH [242]. Furthermore, compared to the minimally invasive rigid channel (needle), soft channel craniopuncture may be an ideal treatment to alleviate cerebral edema after ICH [243]. More studies are needed to determine further and compare the efficacy of craniotomy and MIS in patients with ICH.

#### 7.2.6. Other Treatments

In addition to the treatments discussed above, ICH has many other potential therapeutic targets. Previous studies have shown that statins have various pharmacological effects. Due to its anti-inflammatory and neuroprotective properties, continuous use after ICH reduced mortality at six months and improved early neurologic function. However, studies on the effect of statins on PHE are currently inconsistent. A study with 125 ICH patients reported a positive association between statin use before ICH and decreased absolute and relative perihematomal edema [244]. Another study with 1,275 patients illustrated that statin initiation after ICH was associated with an increase in peak PHE (*β* = 0.12, SE = 0.06, *P* = 0.008). In contrast, continuation versus discontinuation of previous statin treatment was not significantly related to the formation of edema (*P* > 0.10) [245]. A protocol has been published to explore the clinical efficacy of pioglitazone in PHE and hematoma resolution has been published, but the final report has not yet been published [246]. For diabetic patients with acute basal ganglia hemorrhage, another study showed that pretreatment with sulfonylureas reduced PHE upon admission [143]. In one study, researchers administered the nonpeptide vasopressin (AVP) receptor antagonist conivaptan to patients but did not explore whether AVP alleviated PHE after ICH [247]. Furthermore, research indicates that repeated remote daily ischemic conditioning for 7 days improved hematoma resolution and reduced rPHE after ICH [248]. Again, although there was recurring evidence of a reduction in therapeutic hypothermia-induced edema, the effects of therapeutic hypothermia on the outcome of patients with ICH were heterogeneous, as it is currently difficult to determine the extent of damage caused by post-ICH fever currently [123, 140]. Therefore, researchers should pay more attention to potential treatment strategies targeting PHE.

Generally, the clinical condition is very complex. Like hypothermia, if the basic theory on the relationship between fever and ICH damage is not well established, further studies on the therapeutic effects of hypothermia on PHE would be futile. Currently, studies on PHE therapy in other areas, including hemostasis and surgical management, may face the same issues. Thus, it is critical to further analyze the reasons for the inconsistent findings in animal and clinical studies in this review. This may help us better understand why the results of some clinical studies do not match expectations. In addition, it may also benefit the further design of clinical trials on the therapeutic values of specific interventions in PHE.

## 8. Conclusions and Future Directions

**Figure 3:**
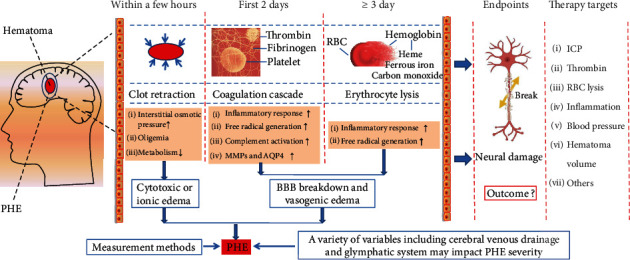
The evolution of PHE and its potential therapeutic targets. Clot retraction, activation of thrombin in the coagulation cascade, and toxicity of RBC degradation products can contribute to PHE formation after acute ICH. Furthermore, abnormal electrolyte and water transport and thrombin and RBC lysis-induced inflammatory and oxidative stress responses are critical in the formation of PHE. PHE can be classified as ionic (cytotoxic) and vasogenic edema according to the mechanisms and imaging characteristics. Although various variables, including cerebral venous drainage and the glymphatic system, can affect the severity of PHE, more accurate methods for quantifying PHE should be developed. PHE can aggravate the severity of brain injury, but the relationship between PHE and functional outcomes after ICH was conflicting. Strategies to alleviate PHE warrant further exploration by targeting ICP, thrombin, lysis of the RBC, inflammation, controlling blood pressure, reducing hematoma volume, etc. Abbreviations: PHE: perihematomal edema; RBC: red blood cell; MMPs: matrix metallopeptidases; AQP4: aquaporin 4; BBB: blood-brain barrier; ICP: intracranial pressure.

ICH is a subtype of stroke with poor outcomes and no effective treatment. Previous studies have explored the underlying mechanisms of PHE by emphasizing the role of the coagulation cascade and hematolysis in the pathological process of ICH. However, there is still a lack of a unified understanding of the classification and staging after ICH. With different limitations or shortcomings, the results of the PHE quantification methods were conflicting. Furthermore, inconsistent results on the relationship between PHE and functional outcomes can limit PHE as a surrogate marker of secondary brain injury or as an endpoint indicator for evaluating the effects of ICH interventions (Figure 3). The formation of PHE requires further investigation at the molecular, cellular, and organ levels. The time phase and imaging characteristics of ionic/cytotoxic and vasogenic edema warrant further exploration after ICH. The acquired knowledge about the mechanisms of PHE formation and the imaging characteristics of PHE will help us identify promising cellular and molecular targets for the treatment of ICH. Deep learning of variables that can influence the evolution of PHE, including sensitive serum biomarkers, will benefit prediction of PHE expansion. Specifically, future research should evaluate the mechanisms of the effects of the cerebral venous drainage system and the brain lymphatic drainage system on PHE.

Furthermore, the quantitative methods of PHE also warrant additional studies because there is still a lack of reliable diagnostic and quantitative assays to evaluate PHE in animals and patients with ICH. Therefore, it is important to evaluate the roles of PHE using the less commonly used MRI in animals with different ICH severities and ICH sites. It is also valuable and necessary to compare the predictive value of PHE in functional outcomes with different PHE measurement protocols in patients. Specifically, it will be of great significance to establish a reliable PHE measurement protocol based on CT images by comparing the standard MRI protocol with CT images that can be easily acquired. Then, more rigorous enrollment criteria (e.g., bleeding in a specific location, hematoma volume within a particular range, and quantification at a particular time point) could be designed to verify the causal relationship between PHE, brain injury severity, and functional outcomes after ICH. Additionally, large prospective multicenter clinical trials could be designed to evaluate the translational importance of novel treatment strategies to alleviate PHE. Further research will help us understand the pathological process of ICH and identify potential therapeutic targets to facilitate translational research.

## 9. Literature Research Strategy

We searched PubMed for articles published in English from January 2017 to February 2022, using the search terms “intracerebral h(a)hemorrhage”, “intracerebral h(a) hemorrhage (o)edema”, “peri-h(a) ematomal (o)edema”, “secondary brain injury”, and “intracerebral h(a)emorrhageury outcomes (prognosis)”. With the term “intracerebral h(a)emorrhage (o)edema”, we found 1091 articles published in the recent five years. 472 articles have been published in the past five years with the terms “intracerebral h(a)emorrhage” and “secondary brain injury”. 136 articles have been published in recent five years with the terms “intracerebral h(a)emorrhage” and “peri-h(a)ematomal (o) edema”. 32 articles published in the last five years with the terms “intracerebral h(a)emorrhage”, “peri-h(a)ematomal (o)edema”, and “secondary brain injury”. 113 articles have been published in the last five years with the terms “intracerebral h(a)emorrhage outcomes (prognosis)” and “peri-h(a)ematomal (o)edema”. Finally, this review includes 151 articles published in the last five years and 108 articles published in the last three years. We also discussed articles published before 2017 if they were considered significant relevant to the scope of this review, especially those on the pathophysiology of PHE and potential therapeutic targets for PHE after ICH. However, we must acknowledge that the limited scope of the search dates for relevant literature on post-ICH edema may bias some areas by omitting relevant older (and newer) literature. In Potential Therapeutic Targets for PHE after ICH, we discuss a large number of treatment interventions. Given the size of this review, we only briefly described the therapeutic effects of dehydration and hypothermia for PHE. Some important messages regarding these may be omitted, and it is worth further searching and evaluating the relevant data.

## Figures and Tables

**Table 1:** The time phase and pathophysiology of PHE.

Time phases	Time points	Events	Main reasons	Mechanisms	PHE classification	References
Hyperacute	Within a few hours	Clot retraction and mass effect	(i) Increase in the interstitial osmotic pressure. (ii) Oligemic and metabolic changes.	(i) Transendothelial Na^+^ gradient. (ii) Extracellular accumulation of neurotoxins (e.g., glutamate).	Cytotoxic or ionic edema	[14, 22–25, 90]

Acute	First 2 days	Coagulation cascade	Activation of thrombin and fibrinogens.	(i) Increase in inflammatory mediators (e.g., TNF-*α*, IL-1*β*, IL-6, IL-12, and ICAMs). (ii) Activation of complement mediators (e.g., C3a and C5a). (iii) Increased expression of matrix metalloproteinases (e.g., MMP-9) and aquaporins (e.g., AQP4). (iv) Inflammatory cell infiltration. (v) Free radical generation.	BBB breakdown and vasogenic edema	[14, 16, 22, 32, 33, 35, 37–40, 43, 44, 47, 48, 51, 52]

Delayed	≥3 days	Erythrocyte lysis	Hemoglobin and its degradation products (e.g., heme, ferric iron, and carbon monoxide).	Increase in the response to oxidative stress and the inflammatory response.	BBB breakdown and vasogenic edema	[2, 14, 22, 57–72]

Abbreviations: PHE: perihematomal edema; TNF-*α*: tumor necrosis factor-*α*; IL-1*β*: interleukin-1*β*; IL-6: interleukin-6; IL-12: interleukin-12; ICAM: intercellular cell adhesion molecules; MMP-9: matrix metalloproteinase-9; AQP4: aquaporin 4; BBB: blood-brain barrier.

**Table 2:** The features of ionic and vasogenic edema in the perihematomal area after ICH.

Classifications	Ionic edema	Vasogenic edema	References
Time phase	Undefined. It may appear in the hyperacute phase or in the late phase of ICH	Undefined. It always appears in the acute and subacute phases of ICH	[25, 77]
Mechanisms	Various ion channels and transporters in the BBB, including Sur1-Trpm4, NKCC1, AQP4, Na^+^-H^+^ exchanger, and Na^+^-Ca^2+^ exchanger, drive this process	Cytotoxic substances such as inflammatory factors and free radical metalloprotease-induced transendothelial permeability pore formation	[79, 98]
Cellular components in the BBB involved	Endothelial cells	Mainly endothelial cells	[79, 98]
BBB integrity	Intact	Partially destroyed (capillaries still maintain BBB structural integrity)	[79, 98]
Reversibility	Reversible	Partially reversible	[79, 98]
Substances transported	Na^+^, Cl^−^, and water	Na^+^, Cl^−^, water, plasma proteins, and other macromolecules (without RBCs)	[14, 22]
Destination of substances transported	From blood vessels to the extracellular space of the brain parenchyma	From blood vessels to the extracellular space of the brain parenchyma	[12, 78, 79, 98]
Imaging characteristics (CT, T1WI, T2WI, and FLAIR images)	It should present as a perihematomal hypodensity seen on CT. Shows an increase in the T2WI and FLAIR image and a decrease in the T1WI	Similar to ionic edema	[82, 84–88]
Imaging characteristics (DWI and ADC images)	The imaging characteristics of ionic edema in DWI and ADC images have not been well defined. If it is similar to cytotoxic edema, it will appear as a reduction in signal on ADC maps but an increase in DWI. However, the chances are high that the imaging features of ionic edema will resemble those of interstitial edema that present as a normal to low signal on DWI and a mild high signal on ADC maps	It shows a normal to low signal on the DWI and a mild high signal on the ADC maps in perihematomal tissue. If the imaging features of ionic edema are finally verified to be similar to those of interstitial edema in perihematomal tissue, it will be challenging to distinguish it from vasogenic edema	[12, 14, 27, 78, 87–89]

Abbreviations: ICH: intracerebral hemorrhage; BBB: blood-brain barrier; Sur1-Trpm4: sulfonylurea receptor 1-transient receptor potential melastatin 4; NKCC1: Na^+^-K^+^-2Cl^−^ cotransporter protein-1; AQP4: aquaporin 4; RBCs: red blood cells; CT: computerized axial tomography; T1WI: T1-weighted MRI; T2WI: T2-weighted MRI; FLAIR: fluid-attenuated inversion recovery image; DWI: diffusion-weighted magnetic resonance imaging; ADC: apparent diffusion coefficient maps.

**Table 3:** The merits and shortcomings of different quantitative methods of PHE after ICH.

Quantitative methods	Merits	Shortcomings	References
*Preclinical studies*			

Brain water content	It is widely used to assess the severity of brain injury in animal ICH models.	It cannot accurately reflect PHE due to the difficulty in separating the perihematomal tissue from normal brain tissue directly.	[9, 101]

Brain swelling	It is a method most used to quantify the extent of brain edema in animals with stable volume of hematoma.	(i) It may be influenced by hematoma volume. (ii) It cannot detect PHE directly and accurately.	[9, 34, 101]

MRI image	MRI may represent the gold standard for detecting and quantifying PHE in animals.	The access of small animals to high-field MRI is limited.	[53, 104–106]

*Clinical studies*			

CT and MRI images	(i) CT images can be easily acquired. (ii) MRI may represent the gold standard for detecting and quantifying PHE in humans. (iii) Based on CT or MRI images, absolute PHE, rPHE, EED, and PHE growth rate have been developed to quantify PHE in humans.	(i) It is challenging to choose suitable thresholds to outline the rim of the PHE. (ii) Absolute PHE, rPHE, EED, and PHE growth rate may be influenced by the quantification time point, the size, shape, and location of the hematoma.	[21, 108–115]

Other methods	Some noninvasive methods including midline shift on CT or MRI images, physical examination, and indirect estimation of ICP may also reflect brain edema after ICH.	They are not sufficient to detect or monitor brain edema and could not directly reflect PHE.	[117–119]

Abbreviations: PHE: perihematomal edema; ICH: intracerebral hemorrhage; CT: computerized axial tomography; MRI: magnetic resonance imaging; EED: edema extension distance; rPHE: relative perihematomal edema; ICP: intracranial pressure.

**Table 4:** Variables that may aggravate PHE in patients.

Baseline variables	Clinical variables	Hematological characteristics	Other clinical variables
Male gender and older age [115]	A higher score on the National Institutes of Health Stroke Scale [115]	Higher platelet count [147]	Cerebral venous drainage system damage (i) AIVF [125] (ii) Negative JVR [157] (iii) Lower CVFV [158]
Genetic characteristics (i) APOE4^+^ [132] (ii) AQP4 (rs1054827) [133] (iii) GC genotype in the TIMP-2-418 position (rs8179090) [134] (iv) Hp 1-1 phenotype [131]	Lower Glasgow Coma Scale score [115]	Systemic inflammatory response (higher neutrophil-lymphocyte ratio) [117, 148]	Glymphatic system damage?
Higher glucose [115]	Larger initial ICH volume [116, 138]	Higher admission hematocrit [21]	
History of hypertension [138]	Irregular hematoma or black hole sign [115, 116]	Higher admission time for partial thromboplastin time [21]	
Higher admission SBP [144, 145]	Larger initial EED [115]	Absence in warfarin preuse [99, 149]	
Impaired blood pressure regulation [139]	Time from symptom onset [138]	Higher serum levels of IL-6 and soluble CD163 [150, 153]	
Higher body temperature [123, 124, 138, 140, 141]	Absence in sulfonylurea drug pretreatment [142, 143]	Higher serum MMP-3 or MMP-8 levels [151, 152]	

Abbreviations: PHE: perihematomal edema; APOE4: apolipoprotein E; AQP4: aquaporin 4; TIMP-2: tissue inhibitor of metalloproteinases 2; Hp: haptoglobin; SBP: systolic blood pressure; ICH: intracerebral hemorrhage; EED: edema extension distance; MMP: matrix metallopeptidase; AIVF: absent in ipsilateral venous filling; JVR: jugular vein reflex; CVFV: cerebral venous outflow volume.

**Table 5:** Clinical studies on the relationship between PHE and neurologic functions after ICH.

Quantitative methods	Study	Design	No. of patients	Functional outcome measures	Imaging modality	Median ICH volume on admission (mL)	Time phase for PHE quantitation	Findings
Absolute PHE volume	Volbers et al. [100]	Retrospective	292	90-day mRS	CT	17.7	Peak PHE volume	The high peak volume of PHE was an independent predictor of the worse outcome on day 90.
	Volbers et al. [20]	Retrospective	220	mRS at discharge	CT	22.8	Within 12 h	The high peak PHE volume predicted a poor discharge outcome.
	Ozdinc et al. [175]	Retrospective	106	30-day mortality	CT	2.14 vs. 18.73 for survivors and nonsurvivors within 30 d after ICH onset	On days 1-12	The absolute area of the perihematomal edema but not the absolute volume of the perihematomal edema was an independent indicator of mortality at 30 days.
	Nawabi et al. [99]	Retrospective	811	90-day mRS	CT	47	Within 12 h	An increase in early PHE volume did not increase the probability of a poor outcome in OAC-ICH but was independently associated with poor outcomes in NON-OAC-ICH.
	Shirazian et al. [176]	Prospective	1,089	30-day mortality, 90-day mRS	CT	22.5	Within 48 h	The absolute increase in PHE within 48 hours after ICH was associated with increased mortality and worse functional outcomes.
	Appelboom et al. [177]	Prospective	133	Discharge outcome (mRS)	CT	Less than 30	Within 24 h	The effect of absolute PHE volume on functional outcome after ICH depended on the size of the hematoma, with only patients with smaller hemorrhages showing poorer results with worse PHE.
	Loan et al. [178]	Prospective	342	Death or dependence (mRS) one year after ICH	CT	48	Within 3 days	The high volume of perihematomal edema did not predict a poor outcome.

PHE growth	Lv et al. [179]	Prospective	233	3-month mRS	CT	13.4	From baseline to 24 hours	Early expansion of PHE was associated with poor outcomes.
	Ye et al. [116]	Prospective	197	90-day mRS	CT	12.7	From baseline to day 3	An increase in PHE volume > 7.98 mL from baseline to day 3 may lead to a poor outcome on day 90 after ICH.
	Grunwald et al. [180]	Retrospective	115	90-day mortality or poor functional outcome (mRS > 2)	CT	11.3 vs. 36.9 for patients with deep and lobar ICH	From baseline to 24 h and 72 h	PHE 72 hours was associated with poor functional outcomes after deep ICH, while PHE 24 hours was associated with mortality for deep and lobar ICH.
	Urday et al. [109]	Retrospective	139	90-day mRS	CT	19	PHE expansion rate between admission and 24-hour post-ICH	A faster PHE expansion rate 24 hours after ICH predicted a worse outcome.
	Murthy et al. [181]	Prospective	596	90-day mRS	CT	15	Within a period of 6 to 72 hours after the onset of ICH	The absolute increase in PHE during the first 72 hours after ICH was associated with worse functional outcomes, particularly with basal ganglia ICH and hematomas < 30 mL.
	Hurford et al. [136]	Prospective	1,028	90-day mRS	CT	13.7	From onset to 72 hours	An increase in EED in the first 72 hours was independently associated with decreased functional outcomes at 90 days.
	Wu et al. [115]	Prospective	861	6-month mortality	CT	14	The first 72 hours	A higher EED than expected was associated with mortality at 6 months.
	Venkatasubramanian et al. [21]	Prospective	27	Barthel index, mRS, and extended GCS scores at 3 months	MRI	33.6	From admission to 48 h	The growth of edema volume was correlated with a decrease in neurologic status at 48 hours, but not with a functional outcome.

rPHE volume	Sykora et al. [139]	Prospective	38	Early neurologic deterioration	CT	20.63	48-72 h after ictus	rPHE independently predicted early neurologic deterioration.
	Arima et al. [138]	Prospective	270	Death or dependency at 90 days	CT	NA	On day 3	Both absolute PHE and rPHE predicted death or dependency at 90 days.
	Gebel et al. [182]	Prospective	142	12-week mRS or 30-day mortality	CT	12.2	Within 3 hours after the onset of ICH and then 1 and 20 hours later	rPHE independently predicted a poor 3-month functional outcome. Absolute edema volume predicted neither mortality nor functional outcome.
	Staykov et al. [110]	Retrospective	219	In-hospital mortality	CT	35.7	Increase in absolute PHE between days 1 and 3, initial rPHE	An increase in absolute PHE but not rPHE between days 1 and 3 was significantly predictive of in-hospital mortality.

Abbreviations: PHE: perihematomal edema; ICH: intracerebral hemorrhage; OAC: oral anticoagulant; NON-OAC-ICH: nonoral anticoagulation-related intracerebral hemorrhage; rPHE: relative perihematomal edema; EED: extension distance; mRS: modified Rankin scale; GCS: Glasgow Coma Scale; CT: computed tomography; MRI: magnetic resonance imaging.

**Table 6:** Preclinical studies on potential therapeutic targets for PHE after ICH.

Potential targets	Authors	Drugs/reagents/treatments	Species	Time points	Main findings	References
Dehydration therapy	Thenuwara et al.	Mannitol, furosemide	Male Sprague-Dawley rats	It was administered intravenously after the baseline measurement of plasma osmolality.	The combination of furosemide with mannitol resulted in a more significant increase in plasma osmolality than seen with mannitol alone and a more significant decrease in brain water at 4 and 8 g/kg of mannitol.	[184]
	Schreibman et al.	Mannitol and hypertonic saline	Male Wistar rats	First, given 5 hours after ICH induction, then administered every 12 hours thereafter (4 doses total).	Increase in plasma osmolarity one hour after infusion. Reduction in mortality and hemispheric swelling at 48 hours. Inhibition in the activation of microglia/macrophages and the infiltration of CD45^+^ cells into perihematomal tissues.	[185]
	Deng et al.	Albumin	Adult male Sprague-Dawley rats	Human serum albumin was administered intravenously one hour after ICH.	Improvement in short- and long-term neurobehavioral deficits. Reduced oxidative stress and neuronal death 72 hours after ICH.	[186]

Inhibition of thrombin and RBC hemolysates	Han et al.	EP3R antagonist AE240	Male C57BL/6 mice	Intraperitoneal injection 20 minutes and 6 hours after striatal thrombin injection and then twice daily for up to 72 hours.	EP3R inhibition mitigated the volume of thrombin-induced brain injury, brain edema, and neurologic deficits.	[32]
	Ye et al.	NA	NA	NA	Thrombin increased blood-brain barrier disruption and brain edema by mediating PAR (PAR-1, PAR-3, and PAR-4) and their downstream signaling.	[37]
	Puech et al.	Dabigatran, rivaroxaban, apixaban, warfarin, and heparin	HBEC-5i human brain endothelial cells	Cells were incubated with or without dabigatran, rivaroxaban, apixaban, warfarin and heparin for 24 hours. The cells were then treated with or without thrombin to mimic a hemorrhagic event for one hour.	Dabigatran treatment allowed the tightness of the endothelial monolayer. Other DOACs limited thrombin-induced alteration of the endothelial monolayer. Pretreatment with warfarin and heparin did not protect against thrombin-induced BBB breakdown. DOACs appear to limit the alteration of BBB in the monolayer of endothelial cells mediated by the thrombin/PAR-1 pathway.	[187]
	Wu et al.	12-(3-Adamantan-1-yl-ureido)-dodecanoic acid (AUDA)	Mouse BV2 microglial and N2A cell lines and C57BL/6 mice.	BV2 microglia were incubated with LPS, thrombin, or hemin in the absence or presence of AUDA for 24 hours. AUDA was administered by intracerebroventricular injection 30 min before ICH.	Reduction in thrombin- and hemin-induced microglial activation *in vitro*. Alleviation in BBB disruption and MMP-9 activity on day 1 after acute ICH *in vivo*.	[188]
	Caliaperumal et al.	Bipyridine	Male Sprague-Dawley rats	ip, 20 mg/kg beginning 6 h post-ICH and then every 24 h for 2 days.	Posttreatment with bipyridine had no impact on nonheme parenchymal iron levels, behavioral impairments, or edema after collagenase-induced ICH. Bipyridine did not reduce tissue loss, cell death, or behavioral impairment in the whole-blood ICH model.	[189]
	Warkentin et al.	Deferoxamine	Male Sprague-Dawley rats	Intraperitoneal administration of DFX at 0 and 6 hours after ICH.	Treatment with DFX treatment does not influence brain edema or functional recovery after ICH.	[190]
	Wu et al.	Deferoxamine (DFX)	C57BL/6 male mice	ip, 6 hours after ICH and then every 12 hours for three days.	DFX did not reduce the volume, edema, or swelling of brain injuries, but improved neurologic function in mice with ICH.	[191]
	Li et al.	VK-28 and deferoxamine (DFX)	C57BL/6 mice	Intraperitoneal administration of VK-28 six hours after ICH and then every 12 hours for one, three, or seven consecutive days.	VK-28 polarized microglia to an M2-like phenotype, reduced brain water content, decreased white matter injury, improved neurologic function, and reduced overall death rate after ICH.	[101]
	Wu et al.	2,2′-Dipyridyl, a lipid-soluble ferrous iron chelator	Male C57BL/6 mice	ip, two hours before collagenase injection or six hours after collagenase or blood injection, and then once daily for 1 or 3 days.	Posttreatment with 2,2′-dipyridyl reduced the volume and edema of the brain injury and improved neurologic function.	[192]
	Zhu et al.	Deferoxamine mesylate- (DFO-) loaded thermosensitive keratin hydrogels (TKG)	Male Sprague-Dawley rats	DFO loaded TKG-3 (20 *μ*L) was injected with a 26 gauge needle into the hematoma core six hours after ICH.	Reduction in ICH-induced iron deposits, brain nonheme iron content, brain edema, and ROS level.	[69]
	Wang et al.	Monascin (a novel natural Nrf2 activator with PPAR*γ* agonist)	Adult male Sprague-Dawley rats	Intragastrical administration of monascin six hours after ICH and twice a day until the euthanasia point.	Alleviation in BBB permeability, edema, and volume of the hematoma.	[71]
	Li et al.	IL-10 or CD36-deficient mice	C57BL/6 male mice, IL-10^−/−^ mice, CD36^−/−^ mice	IL-10 or CD36-deficient mice.	IL-10 accelerated hematoma clearance, alleviated brain water content, and promoted functional recovery as long as CD36 expression was intact.	[57]

Inhibition or modulation of the inflammatory response	Lin et al.	Heme	C57BL/6, mice, TLR4^−/−^ mice	Injection into the striatum.	TLR4^−/−^ mice showed reduced cerebral edema and lower neurological deficit scores.	[194]
	Lai et al.	Verbascoside	Male C57BL/6 mice	Intraperitoneal injection of verbascoside at 15 minutes post-ICH.	Improvement in the behavioral score. Reduction in hematoma volume, brain edema, and neuronal apoptosis by targeting TLR4 in a murine model of acute ICH.	[195]
	Wang et al.	TAK-242	C57BL/6 male mice	Intraperitoneal administration of TAK-242 6 hours after ICH once daily for 5 days.	Reduction in brain water content, neurological deficit scores, and levels of inflammatory factors.	[196]
	Masada et al.	Interleukin-1 receptor antagonist	Male Sprague-Dawley rats	Lateral ventricle injection of ten microliters of adenoviral suspension containing 10^12^ particles/mL immediately after ICH or thrombin injection.	Reduction of polymorphonuclear leukocyte (PMNL) infiltration and brain water content.	[197]
	Rynkowski et al.	C3aRA	Adult male C57BL/6J mice	Intraperitoneal administration of C3aRA 45 minutes before ICH or 6 and 12 hours after ICH, followed in both cohorts by doses twice daily for 72 hours.	Improvement in the neurologic outcome. Reduction in infiltration of inflammatory cells and formation of brain edema.	[198]
	Garrett et al.	C5aRA and C3aRA	Adult male C57BL/6J mice	Intraperitoneal administration of C5aRA and C3aRA 6 and 12 hours after ICH, followed by doses twice daily for 72 hours.	Reduction in brain edema and alleviation of neurologic deficits.	[199]
	Jing et al.	CD47 blocking antibody	Male and female C57BL/6 mice.	Injection of anti-CD47 antibody in 30 *μ*L blood plus 5 *μ*L clodronate or control liposome into the striatum.	Increase in hematoma/iron clearance by macrophages/microglia. Reduction in ICH-induced brain swelling, neuronal loss, and neurological deficits.	[104]
	Rolland et al.	Fingolimod (FTY-720)	Eight-week-old CD-1 mice	Intraperitoneal administration of FTY720 one hour after ICH.	Reduction in brain edema. Improvement in neurological function at all time points tested.	[200]
	Bobinger et al.	Siponimod (BAF-312)	Adult male C57BL/6 mice	Intraperitoneal administration of BAF-312 30 minutes, 24 hours, and 48 hours after ICH.	Siponimod (BAF-312) attenuated PHE after ICH, increased survival, and reduced ICH-induced sensorimotor deficits in the experimental ICH model.	[107]
	Bobinger et al.	Siponimod	C57BL/6N mice	A single (30-minute post-ICH) or multiple (three times: 30 minutes, 24 and 48 hours post-ICH) siponimod administration.	Attenuation in the development of brain edema decreased in ICH-induced ventriculomegaly. Improvement in neurological functions.	[201]
	Xu et al.	CAY10444 (S1PR3 antagonist)	Adult male Sprague-Dawley rats	Administration at 6 hours after ICH and every 24 hours beginning on the second day after ICH.	Modulation of S1PR3 can maintain the integrity of BBB integrity by inhibiting the S1PR3/CCL2 axis after ICH.	[202]
	Wang et al.	Minocycline	Adult male Sprague–Dawley rats	Intraperitoneal administration immediately and 12 hours after ICH, followed by twice a day for two days.	Improvement in the consequences of ICH by preserving the integrity of the BBB. Attenuating neurologic deficits in a manner related to DKK1 involves enhancing Wnt1-*β*-catenin activity.	[36]
	Yang et al.	Minocycline	Male piglets	Intramuscular administration 2 hours after ICH and every 12 hours for 3 days.	Reduction in ICH-induced brain swelling, fewer neurological deficits, and fewer white matter injuries.	[205]

Other therapy	Kim et al.	Pioglitazone	C57 BL/6 mice	Intraperitoneal administration days 1, 3, and 6 after ICH.	Decrease in NLRP3-related brain edema.	[206]
	Wu et al.	Rosiglitazone	Male C57/BL mice	Intraperitoneal administration on days 1, 3, and 6 days after ICH.	Increase in PPAR*γ*, decrease in glutamate, BBB permeability, and neurologic deficit scores.	[207]
	Guo et al.	Nicardipine hydrochloride entrapped in chitosan nanoparticles	Adult male Sprague-Dawley rats	Intranasal administration immediately following ICH.	Reduction in brain water content.	[208]
	Gu et al.	Simvastatin	CD-1 mice	Intragastric administration once a day immediately following ICH.	Reduction in brain edema and cellular apoptosis. Suppression in the NF-*κ*B-mediated MyD88/TRIF signaling pathway.	[209]
	Jiang et al.	Glibenclamide	Sprague-Dawley rats	Glibenclamide was administered intraperitoneally in a single loading dose (10 *μ*g/kg) plus continuous subcutaneous infusion (200 ng/h).	Glibenclamide improved ICH-induced neuroinflammation and improved neurological outcomes in aged rats.	[210]
	Kung et al. and Wilkinson et al.	Glibenclamide	Sprague-Dawley rats	Glibenclamide was administered intraperitoneally in a single loading dose (10 *μ*g/kg) plus continuous subcutaneous infusion (200 ng/h).	Glibenclamide has no influence on hematoma volume, brain water content, and functional impairment in mice with ICH.	[211, 212]
	Wilkinson et al.	Bumetanide, a specific NKCC1 antagonist	Sprague-Dawley rats	Bumetanide ranged from 10 mg/kg to 40 mg/kg was administered orally at 2 hours or 7 days post-ICH.	Bumetanide did not consistently reduce edema, although there was some indication that it may have modest effects after ICH.	[213]
	Li et al.	Neuroserpin	C57BL/6J male mice	Stereotactic injection into the shallow cortex of the hematoma immediately after ICH.	Reduction in brain edema and BBB permeability. Amelioration of neurological deficits.	[214]
	Zhang et al.	Adipose-derived mesenchymal stromal cells (ADSC)	Sprague-Dawley rats	Stereotactic injection into the hemorrhagic brain 48 hours after ICH.	Alleviation in nervous tissue injury and reduction in cell apoptosis. Alleviating brain edema and inhibiting inflammation and expression of the AQP4 protein.	[215]
	Cui et al.	rTMS	C57BL/6J male mice	rTMS was performed every 24 hours for 5 days.	Alleviation of brain edema and functional neural deficits.	[216]
	Baker et al.	Therapeutic hypothermia	NA	The cooling strategies employed in the preclinical studies were highly diverse.	Most studies in ICH animals have shown a significant benefit in behavioral scores, cerebral edema, and the blood-brain barrier. Its usage warrants further exploration.	[140]

Abbreviations: ICH: intracerebral hemorrhage; PAR: protease activated receptors; BBB: blood-brain barrier; DOC: direct oral anticoagulants; S1PR3: sphingosine-1-phosphate receptor 3; DKK-1: dickkopf1; TLR-4: Toll-like receptor 4; CCL2: monocyte chemotactic protein; NF-*κ*B: nuclear factor kappa-B; MyD88: myeloid differentiation factor88; TRIF: adaptor-inducing interferon-*β*; PPAR*γ*: peroxisome proliferator-activated receptor *γ*; AQP-4: aquaporin 4; NLRP3: nucleotide-binding oligomerization domain-like receptor family pyrin domain protein 3; rTMS: repetitive transcranial magnetic stimulation.

**Table 7:** Clinical studies on potential therapeutic targets for PHE after ICH.

Potential targets	Authors	Drugs/reagents/treatments	Samples	Time points	Main findings	References
Dehydration therapy	Shoamanesh et al.	Hypertonic normal saline, mannitol, glycerin, fructose, and albumin	Multiple studies	Guidelines based on multiple studies in the acute phase of ICH	Currently, there is insufficient evidence for the routine or prophylactic use of hyperosmotic agents in ICH. Mannitol and 3% normal saline can be considered a temporizing measure to decrease ICP in patients with ICH with clinical signs of herniation before surgical intervention.	[3]
	Cook et al.	Different dehydrate agents	Multiple studies	Guidelines based on multiple studies in the acute phase of ICH	The use of corticosteroids to treat ICP in ICH can cause harm, has no proven benefits, and is therefore not recommended. There is a dire need for high-quality research to better inform clinicians about the best options for personalized care of patients with cerebral edema.	[221]

Blood pressure control	Leasure et al.	Nicardipine	One thousand patients with primary ICH less than 60 mL and elevated systolic BP (>180 mm Hg)	Within 4.5 hours of onset (target SBP 110–139 mm Hg within 2 hours) or standard (target SBP 140–179 mm Hg within 2 hours)	Decrease in 24-hour PHER in deep ICH. Higher PHER predicted a poor outcome in basal ganglia ICH but not all deep ICH.	[144]
	Zang et al.	Urapidil	121 patients	Within 6 hours	Reduction in rebleeding and PHE. Improvement in short-term quality of life. Early intensive antihypertensive treatment had no impact on mortality.	[225]
	Zhang et al.	Angiotensin-converting enzyme inhibitors and angiotensin II receptor blockers	635 patients	Preuse	Decrease in mortality, volume of perihematomal edema, and prevalence of ICH-associated pneumonia.	[226]

Hemostasis	Jiang et al.	NA	NA	NA	Studies on the efficacy of hematoma expansion are currently controversial. No study has investigated the impact of hemostasis on PHE after ICH.	[227]
	Selim et al.	Deferoxamine mesylate (DFO)	294 patients	DFO (32 mg/kg/day) or saline (placebo) was infused for 3 consecutive days within 24 hours after ICH	Although DFO treatment was safe, it did not change functional outcome on day 90 after ICH.	[231]
	Wei et al.	Deferoxamine mesylate (DFO)	294 patients	DFO (32 mg/kg/day) or saline (placebo) was infused for 3 consecutive days within 24 hours after ICH	In 114 patients with moderate volume of hematoma (10-30 mL), deferoxamine alleviated functional deficit on day 90 after ICH.	[232]

Immunotherapies	Fu et al.	Fingolimod	23 primary supratentorial ICH patients with a volume of hematoma of 5 to 30 mL	Oral administration of fingolimod in 72 hours 3 times	Reduction in PHE. Attenuation in neurologic deficits.	[233]
	Chang et al.	Minocycline	20 patients	Minocycline was administered intravenously once a day, five days in total	No changes in clinical and radiological results. Serum levels of MMP-9 on day 5 tended to be lower in the minocycline group.	[235]
	Fouda et al.	Minocycline	16 patients	Intravenous administration once followed by oral administration every 4 days	Did not influence inflammatory biomarkers, hematoma volume, or perihematomal edema.	[236]

Surgical management	Zuo et al.	Gross-total removal of the hematoma	176 patients with hypertensive basal ganglia hemorrhage	Within 3 hours	Decrease in the formation of perihematomal edema and secondary injury.	[237]
	Fung et al.	Decompressive craniectomy	25 patients	15 (4–69) hours	A noticeable increase in perihematomal edema.	[239]
	Okuda et al.	Craniotomy or stereotactic hematoma evacuation	16 patients	Within 24 hours of onset	Reduction of brain edema.	[238]
	Horowitz et al.	Minimally invasive surgery (MIS)	36 patients	Unavailable	Decrease in pericavity edema.	[241]
	Lian et al.	Minimally invasive surgery+rt-PA	43 patients	6-72 hours after the onset of ICH	MIS reduced PHE volume. rt-PA accelerated clot removal and had no effects on PHE formation. MIS aspiration and a low dose of rt-PA reduced 30-day mortality in patients with severe ICH.	[242]
	Xia et al.	Minimally invasive craniopuncture with the hard- or soft-channel	150 patients with hypertensive intracerebral hemorrhage	Within 24 hours of onset	Minimally invasive soft-channel craniopuncture is more effective in treating HICH in alleviating cerebral edema, inhibiting oxidative stress, and inflammatory response.	[243]

Other therapy	Naval et al.	Statins	125 ICH patients	Prior statin exposure	The mean relative perihematomal edema was significantly lower in patients taking statins at presentation than in patients without prior statin use.	[244]
	Sprugel et al.	Statins	1,275 ICH patients	Initiation of statins after ICH during the first days after ICH	The initiation of statins during the first days after ICH may increase PHE. However, statins should be initiated after that (e.g., at hospital discharge) to prevent cardiovascular events and potentially improve functional recovery.	[245]
	Zhang et al.	Sulfonylureas	27 patients with acute basal ganglia hemorrhage	Sulfonylurea pretreatment before the onset of ICH	The pretreatment of sulfonylureas significantly reduced both PHE volume and rPHE.	[143]
	Corry et al.	Conivaptan	Seven patients	Administered every 12 hours for two days	Conivaptan can be administered safely to ICH patients. Although it evaluated the change in PHE, it did not compare patients without conivaptan.	[247]
	Zhao et al.	Remote ischemic conditioning	40 subjects with supratentorial ICH	Consecutive seven days after ICH	Improvement in the resolution rate of the hematoma and reduction in the relative PHE.	[248]
	Baker et al.	Therapeutic hypothermia	Multiple studies	Systematic review and meta-analysis	It is currently difficult to determine the extent of the harm caused by post-ICH fever. The cooling strategies used in the clinical studies were highly diverse. Definitive randomized controlled studies are still required to answer the therapeutic effects of hypothermia on PHE in ICH patients.	[140]

Abbreviations: ICH: intracerebral hemorrhage; ICP: intracranial pressure; PHE: perihematomal edema; PHER: expansion rate of perihematomal edema expansion rate; BP: blood pressure; SBP: systolic blood pressure; MMP-9: matrix metallopeptidase-9; rt-PA: recombinant tissue plasminogen activator; HICH: hypertensive intracerebral hemorrhage.

## Data Availability

All relevant data for the five patients in Figure 1 are available upon request from the corresponding author. No other datasets were generated or analyzed in this review.

## Abbreviations

ICH: Intracerebral hemorrhage
PHE: Perihematomal edema
BBB: Blood-brain barrier
MRI: Magnetic resonance imaging
ADC: Apparent diffusion coefficient
PARs: Protease-activated receptors
MMP-9: Metalloprotein-9
AQP4: Aquaporin 4
VEGF: Vascular endothelial growth factor
MAC: Membrane attack complex
HO: Heme oxygenase
NKCC1: Na^+^-K^+^-2Cl^−^ cotransporter
KCC: K^+^/Cl^−^ cotransporters
FLAIR: Fluid-attenuated inversion recovery
T1WI: T1-weighted MRI
RBC: Red blood cell
rPHE: Relative PHE
EED: Edema extension distance
ICP: Intracranial pressure
SBP: Systolic blood pressure
APOE: Apolipoprotein E
TIMP-2: Tissue inhibitor of metalloproteinases 2
DOACs: Direct oral anticoagulants
DFX: Deferoxamine
VK-28: 5-[4-(2-Hydroxyethyl) piperazine-1-ylmethyl]-quinoline-8-ol
DFO: Deferoxamine mesylate
TKG: Thermosensitive keratin hydrogels
ROS: Reactive oxygen species
Nrf2: NF-E2-related factor 2
PPAR*γ*: Peroxisome proliferator-activated receptor *γ*
S1PR: Sphingosine 1-phosphate receptor
TLR-4: Toll-like receptor 4
rTMS: Repetitive transcranial magnetic stimulation
ADSC: Adipose-derived mesenchymal stem cells
PHER: Perihematomal edema expansion rate
MIS: Minimally invasive surgery
rtPA: Recombinant human tissue-type plasminogen activator.
